# Attenuation of a Pathogenic *Mycoplasma* Strain by Modification of the *obg* Gene by Using Synthetic Biology Approaches

**DOI:** 10.1128/mSphere.00030-19

**Published:** 2019-05-22

**Authors:** Carole Lartigue, Yanina Valverde Timana, Fabien Labroussaa, Elise Schieck, Anne Liljander, Flavio Sacchini, Horst Posthaus, Brigitte Batailler, Pascal Sirand-Pugnet, Sanjay Vashee, Joerg Jores, Alain Blanchard

**Affiliations:** a INRA, UMR 1332 de Biologie du Fruit et Pathologie, Villenave d’Ornon, France; b Université de Bordeaux, UMR 1332 de Biologie du Fruit et Pathologie, Villenave d’Ornon, France; c Institute of Veterinary Bacteriology, University of Bern, Bern, Switzerland; d International Livestock Research Institute, Nairobi, Kenya; e Institute of Veterinary Pathology, University of Bern, Bern, Switzerland; f J. Craig Venter Institute, Rockville, Maryland, USA; University of Florida

**Keywords:** GTPase Obg, *Mycoplasma*, *Saccharomyces cerevisiae*, attenuated strain, genome engineering, genome transplantation, temperature sensitivity, vaccines

## Abstract

*Mycoplasma* species are responsible for several economically significant livestock diseases for which there is a need for new and improved vaccines. Most of the existing mycoplasma vaccines are attenuated strains that have been empirically obtained by serial passages or by chemical mutagenesis. The recent development of synthetic biology approaches has opened the way for the engineering of live mycoplasma vaccines. Using these tools, the essential GTPase-encoding gene *obg* was modified directly on the Mycoplasma mycoides subsp. *capri* genome cloned in yeast, reproducing mutations suspected to induce a temperature-sensitive (TS^+^) phenotype. After transplantation of modified genomes into a recipient cell, the phenotype of the resulting M. mycoides subsp. *capri* mutants was characterized. Single-point *obg* mutations did not result in a strong TS^+^ phenotype in M. mycoides subsp. *capri*, but a clone presenting three *obg* mutations was shown to grow with difficulty at temperatures of ≥40°C. This particular mutant was then tested in a caprine septicemia model of M. mycoides subsp. *capri* infection. Five out of eight goats infected with the parental strain had to be euthanized, in contrast to one out of eight goats infected with the *obg* mutant, demonstrating an attenuation of virulence in the mutant. Moreover, the strain isolated from the euthanized animal in the group infected with the *obg* mutant was shown to carry a reversion in the *obg* gene associated with the loss of the TS^+^ phenotype. This study demonstrates the feasibility of building attenuated strains of mycoplasma that could contribute to the design of novel vaccines with improved safety.

**IMPORTANCE** Animal diseases due to mycoplasmas are a major cause of morbidity and mortality associated with economic losses for farmers all over the world. Currently used mycoplasma vaccines exhibit several drawbacks, including low efficacy, short time of protection, adverse reactions, and difficulty in differentiating infected from vaccinated animals. Therefore, there is a need for improved vaccines to control animal mycoplasmoses. Here, we used genome engineering tools derived from synthetic biology approaches to produce targeted mutations in the essential GTPase-encoding *obg* gene of Mycoplasma mycoides subsp. *capri*. Some of the resulting mutants exhibited a marked temperature-sensitive phenotype. The virulence of one of the *obg* mutants was evaluated in a caprine septicemia model and found to be strongly reduced. Although the *obg* mutant reverted to a virulent phenotype in one infected animal, we believe that these results contribute to a strategy that should help in building new vaccines against animal mycoplasmoses.

## Abstract (precis)

Animal diseases due to mycoplasmas are a major cause of morbidity and mortality associated with economic losses for farmers all over the world. Currently used mycoplasma vaccines exhibit several drawbacks, including low efficacy, short time of protection, adverse reactions, and difficulty in differentiating infected from vaccinated animals. Therefore, there is a need for improved vaccines to control animal mycoplasmoses. Here, we used genome engineering tools derived from synthetic biology approaches to produce targeted mutations in the essential GTPase-encoding *obg* gene of Mycoplasma mycoides subsp. *capri*. Some of the resulting mutants exhibited a marked temperature-sensitive phenotype. The virulence of one of the *obg* mutants was evaluated in a caprine septicemia model and found to be strongly reduced. Although the *obg* mutant reverted to a virulent phenotype in one infected animal, we believe that these results contribute to a strategy that should help in building new vaccines against animal mycoplasmoses.

## INTRODUCTION

Mycoplasma diseases continue to be a major problem in the livestock sector worldwide. Among the most serious diseases, one can cite contagious bovine and caprine pleuropneumonia, contagious agalactia in small ruminants, enzootic pneumonia in pigs, and chronic respiratory disease in poultry. The socioeconomic cost of these infectious diseases is due mostly to mortality, decreased productivity, trade restrictions, and the costs associated with disease control. The measures currently being pursued for curbing mycoplasma disease mainly rely on antibiotic treatments and prophylaxis, including vaccines if available. Vaccines constitute the most cost-effective measure to control livestock mycoplasma diseases, especially considering increasing antimicrobial resistance, as reported for *Mycoplasma* (1) and other bacteria.

Mycoplasma vaccines have been used for decades, but little progress has been made in developing vaccines that are more efficient and induce long-lasting immunity. Most commercially available mycoplasma vaccines are still made of attenuated mycoplasma strains, obtained after serial passaging of the pathogen, and are generally characterized by their lack of efficacy and the production of adverse reactions (for a review, see reference 2). This is particularly true for ruminant diseases caused by *Mycoplasma* species belonging to the Mycoplasma mycoides cluster (3). Recently, temperature-sensitive (TS^+^) mutants have been widely used for the control of mycoplasmoses in poultry. Most of these vaccines were initially obtained by empirical chemical mutagenesis. This is the case for the attenuated strain of Mycoplasma synoviae, MS-H, that has been used as a commercial poultry vaccine (Vaxsafe MS; Bioproperties Pty. Ltd., Ringwood, Victoria, Australia) in several countries (4). The TS^+^ phenotype has been proposed as a general strategy to design live vaccines (for a review, see reference 5). Interestingly, a genome comparison between M. synoviae strains has indicated that the temperature sensitivity of MS-H is associated with a single-nucleotide change resulting in a Gly123Arg substitution in the Obg sequence (6). The *obg* gene encodes an essential P-loop GTPase in all bacteria. The Obg protein (*Spo0B*-associated GTP-binding protein) acts as a sensor of the GDP/GTP pools and participates in central processes that affect essential cellular functions (7, 8). Recent studies also suggest a role connecting ribosome assembly with the stress response, since it was shown in Escherichia coli that Obg interacts with both the 50S ribosomal subunit and the stringent response alarmone (p)ppGpp (guanosine penta/tetraphosphate) (9).

The direct implication of *obg* mutations in the TS^+^ phenotype in *Mycoplasma* species, including in M. synoviae, has not yet been completely demonstrated (10), as there is a lack of genetic tools for producing mutants. However, other studies conducted using model organisms such as Escherichia coli (8) and Bacillus subtilis (11) revealed that mutations of the Obg protein have been associated with a TS^+^ phenotype. The Obg protein structure has been determined for B. subtilis (12) and for Thermus thermophilus (13). Interestingly, almost all mutations identified so far, associated with a TS^+^ phenotype, have been located in the N-terminal part of the Obg protein (8, 14).

These results, combined with the universality of Obg in bacteria, reinforce the emergence of new therapeutic strategies targeting this essential bacterial GTPase (15). In this study, we used synthetic biology approaches to generate a series of targeted mutations in the *obg* gene of Mycoplasma mycoides subsp. *capri* strain GM12. These changes comprised the mutations known to induce a TS^+^ phenotype in both E. coli and B. subtilis as well as the mutation suspected of being involved in the TS^+^ phenotype of the M. synoviae vaccine strain. The temperature sensitivity of the resulting M. mycoides subsp. *capri* mutants was characterized *in vitro*. A mutant that showed the strongest TS^+^ phenotype was selected and finally tested to evaluate its effect on virulence and pathogenicity using a caprine challenge model. Our results strongly suggest that this mutant is attenuated and demonstrate the feasibility of using synthetic biology approaches to design attenuated next-generation vaccines that express their normal repertoire of antigens and are safe.

## RESULTS

### Selection of the targeted mutations in the M. mycoides subsp. *capri obg* gene.

**FIG 1:**
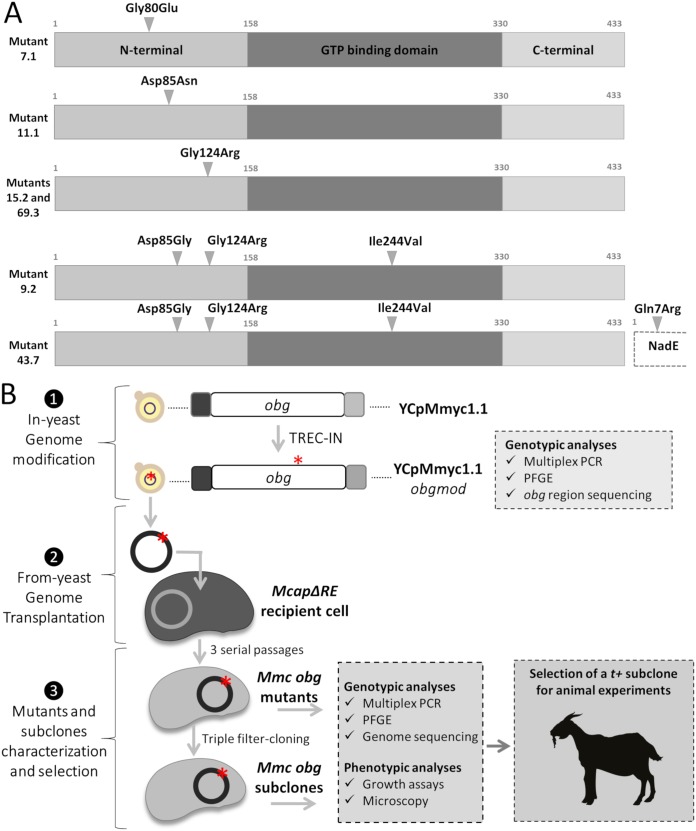
Global strategy used to produce M. mycoides subsp. *capri obg* mutants. (A) Residues targeted in the M. mycoides subsp. *capri* Obg protein during this study. The amino acid changes targeted in this study (Gly80Glu, Asp85Asn, and Gly124Arg) were all located in the N-terminal end of the Obg protein. The Gly80Glu (mutant 7.1) and Asp85Asn (mutant 11.1) substitutions were produced using the nucleotide changes GGT>GAG and GAT>AAC, respectively. The Gly124Arg substitution was produced using two different changes in the nucleotide sequence, either GGG>AGA (mutant 15.2) or GGG>CGT (mutant 69.3). Unexpectedly, one clone (43.7) from the plate for obtaining the Gly124Arg (GGG>AGA) substitution showed three other mutations, two in the *obg* gene, Ile244Val (TAT>TGT) and Asp85Gly (GAT>GGT), and one in the neighboring *nadE* gene, Gln7Arg (CAA>CGA). The latter mutation was repaired and resulted in mutant 9.2. (B) Scheme of the general experimental strategy followed in this study. The YCpMmyc1.1 genome was previously cloned into the yeast strain VL6-48N (7). The replacement of the essential *obg* gene in the YCpMmyc1.1 genome was achieved using TREC-IN, a three-step method allowing the seamless knock-in of the target gene(s) on bacterial genomes cloned into yeast (10). After modification, M. mycoides subsp. *capri* (*Mmc*) *obg-*mutated genomes (YCpMmyc1.1-*obgmod*) were isolated from yeast and transplanted into restriction-free M. capricolum subsp. *capricolum* recipient cells (McapΔRE) to obtain M. mycoides subsp. *capri obg* mutants characterized by mutation at a precise position within the *obg* gene. Mutants and filter-cloned derivatives were characterized *in vitro* (genotypic and phenotypic analyses). The M. mycoides subsp. *capri obg* subclone that showed the most temperature-sensitive phenotype (*t*^+^) was selected for animal experiments using goats.

The protein encoded by the *obg* gene is essential for cell viability in all bacterial species tested so far (16). For mycoplasma species, this essentiality was confirmed in M. pulmonis (17), M. genitalium (18), and M. pneumoniae (19). In the synthetic version of the M. mycoides subsp. *capri* genome constructed at the J. Craig Venter Institute (JCVI-syn1.0), the *obg* gene was shown to be essential in global transposon mutagenesis assays and was retained in the half-reduced genome of the derivative JCVI-syn3.0 (20). The *obg* gene in the genome of M. mycoides subsp. *capri* strain GM12 encodes a 433-amino-acid (aa) protein structured into three domains (see Fig. S1A in the supplemental material). An alignment of the entire M. mycoides subsp. *capri* Obg amino acid sequence with other bacterial Obg protein sequences shows only moderate conservation, with identity percentages ranging from 44.9% with M. synoviae Obg to 41.8% with E. coli and B. subtilis Obg and 37.7% with T. thermophilus Obg (Fig. S1B). However, the identity scores increase up to ∼50% with M. synoviae and B. subtilis Obg proteins when considering the Obg fold and GTP-binding domains only (Fig. S1B). In M. synoviae, the mutation that has been associated with a TS^+^ phenotype is a Gly123Arg substitution (6). We chose to produce the same mutation in the M. mycoides subsp. *capri obg* gene using two different changes in the nucleotide sequence: either GGG>AGA or GGG>CGT. In addition, we produced the mutations Gly80Glu (GGT>GAG) and Asp85Asn (GAT>AAC) that have been reported to be associated with a TS^+^ phenotype in B. subtilis (Gly80Glu and Asp85Asn) (11), E. coli (Gly80Glu and Asp85Asn) (8), and Caulobacter crescentus (Gly80Glu only) (21) (Fig. 1A). We included two nucleotide changes per codon to limit the possibility of reversion during both the *in vitro* and *in vivo* cell propagation. It should be noted that even though the targeted Gly123, Gly80, and Asp85 residues are conserved between M. mycoides subsp. *capri*, M. synoviae, B. subtilis, and E. coli, the amino acid numbering slightly differs from one organism to another. Throughout this text, the numbering of the positions of the residues in the M. mycoides subsp. *capri* Obg sequence was adopted to avoid confusion.

10.1128/mSphere.00030-19.2 FIG S1 Comparison of Obg sequences from Bacillus subtilis (Bsub), Escherichia coli (Ecoli), Thermus thermophilus (Ttherm), Mycoplasma synoviae (MsTs−), the Mycoplasma synoviae vaccine strain (MsTs+), and Mycoplasma mycoides subsp. *capri* (*Mmc*). (A) Multiple Obg sequence alignment generated using ClustalW. Residues targeted in this study are highlighted in blue (positions 80, 85, and 124). They are all found in the glycine-rich N-terminal domain of the protein. The additional mutation found in mutants 9.2 and 43.7 is highlighted in green in the GTP-binding domain of the protein. This domain contains the conserved G motifs (G1 to G5) responsible for GDP and GTP binding as well as the switch I and II domains. These regions are known to mediate the conformational switch between the GDP- and GTP-bound forms of the Obg protein. The C-terminal domain of the protein is not conserved. (B) Percent identities between M. mycoides subsp. *capri* Obg and other Obg proteins (whole sequence or part of the protein). Download FIG S1, PDF file, 0.6 MB. Copyright © 2019 Lartigue et al. 2019 Lartigue et al. This content is distributed under the terms of the Creative Commons Attribution 4.0 International license.

### Generation of M. mycoides subsp. *capri obg* mutants.

Figure 1B shows the general experimental strategy conducted in this study that led to the generation of the different TS^+^ M. mycoides subsp. *capri* mutants as well as the different validation methods used at each step. Each of the above-mentioned substitutions was introduced into the parental M. mycoides subsp. *capri* genome (strain GM12) formerly cloned into yeast (YCpMmyc1.1) (22), using the TREC-IN (tandem repeat endonuclease cleavage-insertion) method, an approach allowing seamless gene replacement in yeast (Fig. 1B, step 1, and Fig. S2). Yeast transformants were screened by PCR, and the amplicons covering the *obg* region were sequenced to confirm the presence of the designed mutations (Fig. S3). One of the yeast transformants (clone 43 [cl43]), isolated initially to obtain the GGG>AGA (Gly124Arg) mutant, carried additional point mutations. Indeed, this particular clone presented four mutations in total, three in the *obg* gene, GAT>GGT (Asp85Gly), GGG>AGA (Gly124Arg), and TAT>TGT (Ile244Val), and one in the downstream *nadE* gene that encodes a putative NH_3_-dependent NAD^+^ synthetase (CAA>CGA [Gln7Arg]). Because of the presence of mutations at two targeted positions (Gly124Arg and Asp85Gly), yeast clone 43 was subjected to further analysis. In order to take into account any potential impact of the substitution located on the *nadE* gene, an additional yeast clone (cl19) was constructed by repairing this substitution (Fig. S4). Genome integrity was verified by multiplex PCR and pulsed-field gel electrophoresis (PFGE) (Fig. S3 and S4), and six yeast clones (cl2, cl37, cl80, cl69, cl43, and cl19) carrying each the modified M. mycoides subsp. *capri* genomes were selected.

10.1128/mSphere.00030-19.3 FIG S2 Production of *obg* mutations using the TREC-IN (tandem repeat endonuclease cleavage-insertion) method and PCR screening of the yeast transformants (36). The genome of the modified M. mycoides subsp. *capri* clone YCpMmyc1.1 (strain GM12) carries a yeast centromere, CEN6; a yeast autonomously replicative origin, ARSH4; the histidine auxotrophic marker for proper propagation in yeast; as well as a tetracycline marker for selection in mycoplasma. The CORE6 mutagenesis cassette was amplified by PCR from plasmid pCORE6 using the chimeric primers core6-obg-F1 and core6-obg-R1 (see Table S1 in the supplemental material) and transformed into the VL6-48N yeast strain harboring the YCpMmyc1.1 genome according to methods described previously by Gietz et al. (40). Yeast transformants were selected on SD-His-Ura plates and screened by PCR using primers Obg-F1, Obg-R1, Obg-F2, and Obg-R2 (Fig. S3). The *obg* mutagenesis cassettes, made of the 3′ end of the kanamycin resistance marker (positions 265 to 810) and the M. mycoides subsp. *capri obg* gene with mutations at position 239/240, 253/255, or 369/371 (corresponding to position 80, 85, or 124 of the Obg protein, respectively), were produced by the assembly of three overlapping minicassettes (minicassettes A, B, and C) (C). Minicassette A, common for all *obg* mutagenesis cassettes, was PCR amplified using the plasmid template pFA6a-kanMX4 (GenBank accession no. AJ002680) and the primer pair cassette A-F1/cassette A-R1 (Table S1). Minicassettes B and C were PCR amplified from WT M. mycoides subsp. *capri* GM12 genomic DNA using the primer pairs cassette B-F1/cassette B-R1 and cassette C-F1/cassette C-R1, respectively. The sequences of the primers cassette B-R1 and cassette C-F1 varied in accordance with the position targeted (position 239/240, 253/255, or 369/371 of the *obg* gene) (Table S1). After the assemblies of the three overlapping minicassettes by the Gibson assembly method (41), the *obg* mutagenesis cassettes were sequenced to ensure their integrities and transformed into VL6-48N yeast clone 34 harboring the YCpMmyc1.1*-Δobg*::*URA3* genome. Yeast transformants were selected on YPDA plates supplemented with Geneticin at 0.2 mg · ml^−1^ and screened by PCR using primers Obg-F1, Obg-F2, and Obg-R2 (Fig. S3). True recombinants (YCpMmyc1.1*-obg*::*URA3*) were plated onto galactose solid medium to induce the expression of I-SceI and a DNA double-strand break at the I-SceI site. Yeast clones that have seamlessly replaced the original *obg* gene with its mutated version (excision of the pgal-ISceI-pURA3-Kan fragment) were counterselected on SD-His plus 5-fluoroorotic acid (5-FOA). Five primers were designed to check the replacement of the CORE6 cassette in the YCpMmyc1.1 genome cloned into yeast (step 1), the insertion of the *obgmod* cassette into the YCpMmyc1.1*-Δobg*::*URA3* genome (step 2), and the excision of the pgal-I-SceI-pURA3-Kan fragment from the YCpMmyc1.1*-obgmod*::*URA3* genome (step 3). (A) Positions of the five primers as well as expected sizes of the amplicons. (B) Primer names and sequences. (C) Assembly of the mutagenesis cassette *obgmod* from the three overlapping minicassettes A, B, and C produced by PCR. The reverse primer used to amplify minicassette B and the forward primer used to amplify minicassette C contained the nucleotides changes (red star) to introduce the desired mutation into the final assembly product. A total of four *obgmod* DNA cassettes were produced, carrying mutations at either position 239/240 (GGT>GAG), position 253/255 (GAT>AAC), or position 369/371 (GGG>AGA or GGG>CGT), which corresponds to position 80, 85, or 124 of the Obg protein, respectively. Gray star, I-SceI restriction site. Download FIG S2, PDF file, 0.4 MB. Copyright © 2019 Lartigue et al. 2019 Lartigue et al. This content is distributed under the terms of the Creative Commons Attribution 4.0 International license.

10.1128/mSphere.00030-19.4 FIG S3 Genotypic analyses of the yeast transformants during the TREC-IN process. Yeast transformants were screened by (i) simplex PCR at steps, 1, 2, and 3 of the TREC-IN process and (ii) multiplex PCR, PFGE, and sequencing of the *obg* gene at the end of the TREC-IN process. (A) The replacement of the original M. mycoides subsp. *capri obg* gene by the CORE6 cassette in the YCpMmyc1.1 genome cloned into yeast VL6-48N clone 7.3 (step 1) was confirmed by the presence of a 3,541-bp instead of a 1,601-bp amplicon using the primers Obg-F1 and ObgR2 (see Fig. S2 in the supplemental material). Eight clones out of 10 showed the correct profile. Yeast clone 34 was selected for pursuing the experiment. (B) Four *obgmod* DNA cassettes carrying mutations at either position 239/240 (GGT>GAG), position 253/255 (GAT>AAC), or position 369/371 (GGG>AGA or GGG>CGT), which corresponds to position 80, 85, or 124 of the Obg protein, respectively, were individually introduced into yeast YCpMmyc1.1*-Δobg*::*URA3* clone 34 (step 2). In all cases, the insertion of the *obgmod* cassette in the YCpMmyc1.1*-Δobg*::*URA3* genome was confirmed by the presence of a 5,402-bp amplicon using the primer pair Obg-F11/ObgR2 or a 2,415-bp amplicon using the primers pair Obg-F2/ObgR2 (Fig. S2). (B) Results obtained only for the *obgmod* DNA cassettes containing the mutations at position 369/371 (GGG>AGA). Here, yeast cl34 was directly transformed with either the three overlapping minicassettes A, B, and C (A+B+C) or the *obgmod* cassette produced by the assembly of minicassettes A, B, and C by Gibson assembly (Fig. S2C). A total of 21 clones were screened with the primers pair Obg-F2/ObgR2; 13 clones showed a band at 2,415 bp, and 8 clones showed a mix of profiles with a band at 2,415 bp and another at 701 bp. The mixed clones made of yeast cells containing an M. mycoides subsp. *capri* genome without *obg* (step 1) or an M. mycoides subsp. *capri* genome with the modified *obg* gene (step 2) were generally kept at this stage. Indeed, the cells that have not integrated the *obgmod* cassette should not be able to survive at step 3 (triple counterselection in 5-FOA). The *obg* region was sequenced with specific *obg* primers to confirm the presence of the desired mutations at the correct position of the gene in the pure clones as well as mixed clones. Sequencing data showed that most of the clones contained the desired mutations, but a few yeast clones contained unexpected mutations in the *obg* gene. Among them, yeast clone 43 containing four mutations (three in the *obg* gene and one in the *nadE* gene) (Table 1) was conserved. Fourteen clones (11 that showed a clean profile in the previous PCR and 3 that showed a mixed profile) were also screened with primer pair Obg-F11/ObgR2; 11 showed the correct 5,402-bp fragment. The same screening was applied for the three other cassettes. (C) Clones selected at the second step of the TREC-IN process were plated for triple counterselection in 5-FOA. The excision of the pgal-I-SceI-pURA3-Kan fragment from the YCpMmyc1.1*-bgmod*::*URA3* genomes (step 3) was evidenced by the amplification of a 1,602-bp fragment using the Obg-F1/ObgR2 primer pair and no amplification with primer pair Obg-F1/ObgR1 or Obg-F2/ObgR2. The *obg* region was sequenced once more to verify the presence of the desired mutations at the correct position of the *obg* gene. Finally, yeast clones 2, 37, 80, and 69, having M. mycoides subsp. *capri* genomes with nucleotide changes at position 239/240 (GGT>GAG), position 253/255 (GAT>AAC), position 369/371 (GGG>CGT), and position 369/371 (GGG>AGA), respectively, were selected, as was yeast clone 43, showing several unexpected mutations in the *obg* region. M, 1-kbp plus DNA ladder from Invitrogen; H, negative control in which the DNA template was replaced by H_2_O; Mc, control in which YCpMyc1.1 genomic DNA was used as the DNA template; T7, control in which the genomic DNA from yeast clone 7 extracted at step 1 of TREC-IN was used as the DNA template; T9, control in which the genomic DNA from yeast clone 9 extracted at step 2 of TREC-IN was used as the DNA template. (D) Selected yeast clones 2, 37, 80, 43, and 69 were then analyzed by multiplex PCR. Eleven pairs of primers producing 11 amplicons from ∼0.1 to ∼1 kbp with ∼0.1-kbp increments were designed every 100 kbp in the M. mycoides subsp. *capri* genome (Table S1) and used in a single PCR mixture with 50 to 100 ng of yeast total DNA template. All selected clones showed the expected 11-band pattern, also found for the M. mycoides subsp. *capri* YCpMyc1.1 genomic DNA control (Mc). Mc, control in which WT M. mycoides subsp. *capri* genomic DNA is used as the DNA template. (E) The integrity of M. mycoides subsp. *capri* engineered genomes was finally verified by pulsed-field gel electrophoresis. Briefly, yeast agarose plugs (from all 5 clones) were prepared using the CHEF mammalian genomic DNA plug kit (Bio-Rad), according to the protocol described by Lartigue et al. (22), and then subjected to electrophoresis in a 1% pulsed-field agarose gel (Bio-Rad) in 1× Tris-acetate-EDTA (TAE), with a contour-clamped homogeneous electric field (CHEF DR III; Bio-Rad). Prior to PFGE, yeast chromosomal DNA was removed from agarose plugs by treatment of the agarose plugs with a cocktail of restriction enzymes (AsiSI, FseI, and RsrII) that have multiple recognition sites in yeast chromosomes and none in the M. mycoides subsp. *capri* genome and subsequent electroremoval of fragmented yeast DNA by agarose electrophoresis. After this step, the circular DNA that remained in agarose plugs was restricted with BssHII and finally separated by PFGE. Pulse times were ramped from 60 to 120 s for 24 h at 6 V · cm^−1^. After electrophoresis, the gel was stained with SYBR gold, and DNA patterns were visualized using the E-Box VX2 gel documentation system. Sizes of restriction fragments are given in kilobases on the right of the gel. Bands of ∼668 and 419 kbp were found in all clones tested. These sizes matched the expected size of the BssHII-restricted YCpMyc1.1 genome. M, yeast chromosome PFGE marker (New England Biolabs) in kilobase pairs; Y, Saccharomyces cerevisiae VL6-48N. Download FIG S3, PDF file, 0.3 MB. Copyright © 2019 Lartigue et al. 2019 Lartigue et al. This content is distributed under the terms of the Creative Commons Attribution 4.0 International license.

10.1128/mSphere.00030-19.5 FIG S4 In-yeast construction of M. mycoides subsp. *capri* genomes carrying mutations at position 254 (GAT>GGT), position 369/371 (GGG>AGA), and position 732 (ATA>GTA) of the *obg* gene and no mutation in the *nadE* gene. (A) PCR results obtained at the third step of the TREC-IN process. Yeast clones showing an amplification of 1,601 bp with primer pair obgF1/R2 and no amplification with primer pairs obgF2/R2 and obgF1/R1 were conserved. T1, control in which the genomic DNA from yeast clone 34 extracted at step 1 of TREC-IN was used as the DNA template; T2, control in which the genomic DNA from yeast clone 83 extracted at step 2 of TREC-IN was used as the DNA template; M, 1-kbp plus DNA ladder from Invitrogen. (B) Multiplex PCR results obtained with 5 out of the 19 positive clones. The five clones showed the expected 11-band pattern as for the M. mycoides subsp. *capri* YCpMyc1.1 control (Mc). H, PCR H_2_O control. (C) M. mycoides subsp. *capri* genomes were finally checked by PFGE. Selected clone 19 showed the two-band profile expected for full-length M. mycoides subsp. *capri* genomes just like the control strain VL6-48N cl7.3 containing the M. mycoides subsp. *capri* YCpMyc1.1 genome. Details about the procedure are provided in the legend of Fig. S3 in the supplemental material. M, yeast chromosome PFGE marker (New England Biolabs) in kilobase pairs; Y, Saccharomyces cerevisiae VL6-48N. At the end of the procedure, yeast clone 19 was selected for the transplantation experiment. Download FIG S4, PDF file, 0.3 MB. Copyright © 2019 Lartigue et al. 2019 Lartigue et al. This content is distributed under the terms of the Creative Commons Attribution 4.0 International license.

10.1128/mSphere.00030-19.9 TABLE S1 PCR primer pairs used during this study. Nucleotide changes compared to the original *obg* sequence are shown with boldface type and underlining. Download Table S1, XLSX file, 0.01 MB. Copyright © 2019 Lartigue et al. 2019 Lartigue et al. This content is distributed under the terms of the Creative Commons Attribution 4.0 International license.

The modified M. mycoides subsp. *capri* genomes were isolated from the yeast clones and independently transplanted into McapΔRE recipient cells (Fig. 1B, step 2). The resulting transplants were serially cultured for three passages, and their genomes were sequenced to confirm the expected substitutions on the *obg* gene. Altogether, six M. mycoides subsp. *capri obg* mutants were finally selected for phenotypic characterization (Fig. 1B, step 3), with four presenting a single-amino-acid substitution (mutants 7.1, 11.1, 15.2, and 69.3) and two presenting multiple substitutions (mutants 43.7 and 9.2) (Fig. 1A and Table 1).

**TABLE 1:** M. mycoides subsp. *capri obg* mutants and subclones obtained in this study^*c*^

M. mycoides subsp. *capri* *obg* mutant	*obg* nucleotide change(s) (position)^*a*^	Obg amino acid change(s)^*a*^	NadE amino acid change	TS phenotype of mutant^*b*^	M. mycoides subsp. *capri* *obg* subclone	TS phenotype of clone^*b*^
11.1	GAT>AAC (253/255)	Asp85Asn		None	ND	ND

15.2	GGG>CGT (369/371)	Gly124Arg		None	ND	ND

69.3	GGG>AGA (369/371)	Gly124Arg		None	ND	ND

7.1	GGT>GAG (239/240)	Gly80Glu		+	7.1.2A	+
					7.1.3A	+

43.7	GAT>GGT (254), GGG>AGA (369/371), ATA>GTA (732)	Asp85Gly, Gly124Arg, Ile244Val	Gln7Arg (CAA>CGA)	+++	43.7.3E	+++
					43.7.4B	+++

9.2	GAT>GGT (254), GGG>AGA (369/371), ATA>GTA (732)	Asp85Gly, Gly124Arg, Ile244Val		+++	9.2.1A	+++
					9.2.3B	+++

a Numbering refers to the nucleotide and amino acid positions in the M. mycoides subsp. *capri obg* gene and protein.

b None, no TS^+^ phenotype; +, weak TS^+^ phenotype; +++, strong TS^+^ phenotype (as determined by culture assays).

c ND, not determined.

### Phenotypic characterization of the M. mycoides subsp. *capri obg* mutants.

**FIG 2:**
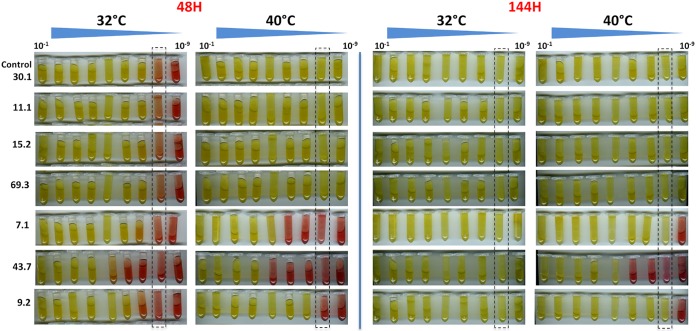
Growth monitoring of M. mycoides subsp. *capri obg* mutants at permissive (32°C) and nonpermissive (40°C) temperatures. M. mycoides subsp. *capri obg* mutants were first precultured in SP5 medium at 32°C. At pH 6.4, cell cultures were then serially diluted (10^−1^ to 10^−9^) and incubated at either 32°C or 40°C for 8 consecutive days. SP5 medium color change from red to yellow evidenced cell propagation, since glucose utilization during M. mycoides subsp. *capri* propagation results in an acid shift. Pictures taken at 48 h and 144 h are shown, as indicated. Control 30.1 corresponds to YCpMmyc1.1 with an intact *obg* region. The dotted rectangles indicate tubes (dilution, 10^−8^) from which the 100-μl aliquots were removed every 24 h from 0 h to 144 h to perform the qPCR analysis (Fig. 3A and B).

The impact of the above-mentioned *obg* substitutions on M. mycoides subsp. *capri* growth was evaluated by combining culture assays and quantitative PCR (qPCR) analyses. All M. mycoides subsp. *capri obg* mutants were cultivated in standard SP5 mycoplasma medium at 32°C and 40°C together with the parental clone 30.1 (YCpMmyc1.1 with a wild-type [WT] *obg* region) as a control. The color change of the medium was first observed daily during 8 consecutive days (48-h and 144-h time points are shown in Fig. 2). Similarly to WT M. mycoides subsp. *capri* strain GM12 (data not shown), the control clone 30.1 grew slightly faster at 40°C than at 32°C and reached a maximum titer (>10^9^ color-changing units [CCU] · ml^−1^) after 48 h of incubation at 40°C. Unexpectedly, the M. mycoides subsp. *capri obg* mutants 11.1, 15.2, and 69.3 with the single-amino-acid changes Asp85Asn (GAT>AAC) and Gly124Arg (GGG>AGA or GGG>CGT) grew as well as the control at both temperatures tested and therefore did not show a TS^+^ phenotype (Fig. 2). In contrast, the mutants (7.1, 43.7, and 9.2) with the single mutation Gly80Glu (GGT>GAG) and with the multiple mutations Asp85Gly (GAT>GGT), Gly124Arg (GGG>AGA), and Ile244Val (TAT>TGT), with or without a mutation in *nadE* (Gln7Arg, CAA>CGA), showed a marked TS^+^ phenotype. Clone 43.7 was the most affected, with a 4-log lag behind the control clone 30.1 still after 144 h of culture at 40°C (Fig. 2).

**FIG 3:**
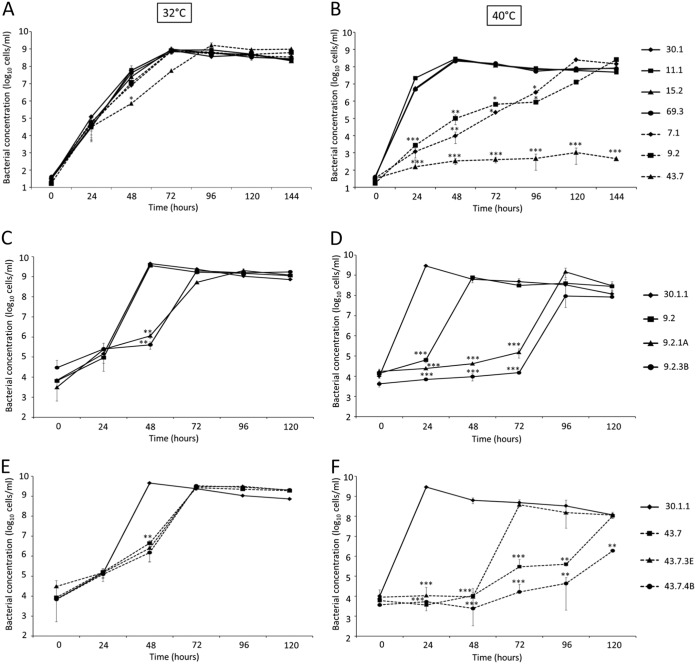
Temperature sensitivity of the different M. mycoides subsp. *capri obg* mutants and subclones as measured by qPCR. Bacterial concentrations of the different M. mycoides subsp. *capri obg* mutants and subclones were measured at 32°C (A, C, and E) and 40°C (B, D, and F) over time. (A and B) Cultures of the different M. mycoides subsp. *capri obg* mutants incubated at 32°C (A) or 40°C (B) were sampled every 24 h for up to 144 h, and bacterial titers were assessed using a qPCR assay targeting the 16S rRNA gene. (C to F) Similar experiments were performed to estimate the growth of individual subclones derived from the initial mutant 9.2 (C and D) or mutant 43.7 (E and F) for up to 120 h. Cultures monitored over time for qPCR assays are surrounded by dotted rectangles (Fig. 2 [for the mutants]; see also Fig. S5 in the supplemental material [for the subclones]). Errors bars represent standard deviations (SD) from three independent replicates. Statistical differences (*t* test) between the M. mycoides subsp. *capri* controls (30.1 or 30.1.1) and the different mutants were determined at each time point. *, *P* < 0.05; **, *P* < 0.001; ***, *P* < 0.0001.

As the color change of the medium does not precisely reflect the cell concentration in a mycoplasma culture, we performed 16S rRNA gene qPCR analyses during the *in vitro* culture. While the qPCR curves confirmed the results obtained by the color change culture assay (Fig. 3), they provided a more precise measure of mycoplasma growth. At 32°C, we observed that the curves were very similar between the M. mycoides subsp. *capri* control clone 30.1 and the different mutants tested (Fig. 3A). All bacterial cultures reached their stationary phase after 72 h of incubation, except for mutant 43.7, which was slightly delayed and reached its stationary phase after 96 h (24 h later). At 40°C (Fig. 3B), no growth difference was observed between mutants 11.1, 15.2, and 69.3 and the control strain 30.1. They all reached the stationary phase at the 48-h time point (*T*_48_), confirming that M. mycoides subsp. *capri* grows faster at 40°C than at 32°C, but an ∼1-log decrease in the final bacterial concentration was consistently measured (∼10^8^ cells · ml^−1^ at 40°C versus ∼10^9^ cells · ml^−1^ at 32°C) (Fig. 3A and B). Interestingly, the growth curves of three other mutants did not follow the trend of the control strain. At 40°C, the doubling time of mutants 7.1 and 9.2 appeared to be greatly affected, since these cells reached the stationary phase at *T*_120_ and *T*_144_, respectively. In addition, mutant 43.7 did not appear to grow at all, since the bacterial concentration remained consistently below 10^3^ cells · ml^−1^ (Fig. 3B), even after 144 h of incubation. However, according to Fig. 2, a color change from red to yellow could be observed for this mutant up to a 10^−5^ dilution, indicating a residual ability to grow. Consistent with this result, the qPCR measurements of the *in vitro* culture done with the 10^−8^ dilution did not reveal any growth (Fig. 2, dotted rectangles). Based on these results, mutants 7.1, 9.2, and 43.7 were selected for further analysis.

10.1128/mSphere.00030-19.6 FIG S5 Growth monitoring of M. mycoides subsp. *capri obg* subclones at permissive (32°C) and nonpermissive (40°C) temperatures over time. M. mycoides subsp. *capri* subclones were first precultured in SP5 medium at 32°C. A pH 6.4, cell cultures were then serially diluted (10^−1^ to 10^−9^) and incubated at either 32°C or 40°C for 8 consecutive days. SP5 medium color change from red to yellow indicated cell propagation, since glucose utilization during M. mycoides subsp. *capri* propagation results in an acid shift. Pictures taken at 48 h and 120 h are shown, as indicated. Subclone 30.1.1 that corresponds to YCpMmyc1.1 with an intact *obg* region served as a control. Dotted rectangles indicate tubes (dilution, 10^−5^) from which the 100-μl aliquots were removed every 24 h from 0 h to 120 h to perform the qPCR analysis (Fig. 3C to F). Download FIG S5, PDF file, 0.5 MB. Copyright © 2019 Lartigue et al. 2019 Lartigue et al. This content is distributed under the terms of the Creative Commons Attribution 4.0 International license.

### Genotypic and phenotypic analyses of the selected M. mycoides subsp. *capri obg* subclones.

In order to eliminate the possibility of having heterogeneous populations, the three mutants (7.1, 9.2, and 43.7) were submitted to three rounds of filter cloning (Fig. 1B). After this procedure, a total of six subclones were selected for each of the three selected mutants and cultured in SP5 medium supplemented with tetracycline prior to genotypic analysis. For mutants 7.1 and 43.7, all subclones showed an *obg* sequence identical to that of the parental clones. However, for mutant 9.2, the sequencing data of the 6 subclones revealed some sequence heterogeneity. While 4 subclones carried the expected the GAT>GGT (Asp85Gly), GGG>AGA (Gly124Arg), and TAT>TGT (Ile244Val) substitutions, 2 others (9.2.2 and 9.2.6) presented a different mutation in one of the targeted codons, GAT>AGT (Asp85Ser). This suggested the coexistence of two populations in the initially selected 9.2 mutant. Consequently, subclones 9.2.2 and 9.2.6 with an unwanted additional mutation were not analyzed further.

Phenotypic analysis of the subclones was performed as described above, using two subclones from each parental mutant (Table 1). After evaluating the temperature sensitivity phenotype of the subclones by evaluating their growth at two temperatures (Fig. S5), data from the phenotypic analysis of the subclones that showed the most pronounced phenotype (43.7.3E, 43.7.4B, 9.2.1A, and 9.2.3B) were confirmed using qPCR assays (Fig. 3C to F). At 32°C, the cultures of the four subclones reached the stationary phase at *T*_72_, showing a 24-h delay compared to the control strain 30.1.1 (Fig. 3C and E). This delay significantly increased at 40°C, a temperature at which almost all subclones reached their optimum growth beyond 96 h of incubation, in contrast to 24 h for the control strain 30.1.1. Only subclone 43.7.3E reached its stationary phase after 72 h of incubation (Fig. 3F). As observed for growth curves, the subclones derived from mutant 9.2 (9.2.1A and 9.2.3B) behaved differently from the parental mutant, as they both grew slow, especially at 40°C (Fig. 3D). In fact, their phenotype became almost identical to that of subclones 43.7.3E and 43.7.4B, which contain the same mutations in the *obg* gene (Asp85Gly [GAT>GGT], Gly124Arg [GGG>AGA], and Ile244Val [TAT>TGT]) plus a mutation in *nadE* (Gln7Arg [CAA>CGA]). This discrepancy, most likely caused by the presence of a mixed population in mutant 9.2, suggested that the mutation GAT>AGT (Asp85Ser) detected in some 9.2 subclones probably does not confer a TS^+^ phenotype.

The culture assays (Fig. S5) together with the qPCR measurements confirmed that subclones 43.7.3E, 43.7.4B, 9.2.1A, and 9.2.3B exhibit a marked TS^+^ phenotype.

Whole-genome sequencing of subclones derived from the 30.1 control and from the 7.1, 9.2, and 43.7 mutants indicated a number of mutations other than those in the *obg* gene, but none of these correlated with the TS^+^ phenotype observed (Table S2).

10.1128/mSphere.00030-19.10 TABLE S2 Genome sequencing and variant analysis of *obg* subclones. After triple filter cloning, individual subclones were grown, and genomic DNA was purified using the Wizard genomic DNA purification kit (Promega). Genome sequencing was performed on an Illumina MiSeq system using paired-end libraries. About 300,000 to 1,500,000 paired reads of 150 nucleotides (nt) were produced for each genome. Data processing, including quality check, trimming, alignment with BWA (Galaxy version 1.2.3), and variant calling using Varscan (Galaxy version 0.1), was completed using Galaxy instance (https://usegalaxy.org) (42). Overall, a total of 25 additional mutations were identified in one or several of the 17 subclones analyzed, in comparison to the 30.1.1 subclones. Sixteen were located in intergenic regions, and nine were located within coding DNA sequences (CDSs). However, the distribution of these mutations did not correlate with the TS^+^ phenotype observed, confirming the impact of the mutations introduced into the *obg* locus. The sequence of the *obgE-nadE* region previously obtained by PCR and Sanger sequencing was also confirmed in all cases. Download Table S2, XLSX file, 0.03 MB. Copyright © 2019 Lartigue et al. 2019 Lartigue et al. This content is distributed under the terms of the Creative Commons Attribution 4.0 International license.

### Evaluation of the virulence of the 9.2.3B *obg* subclone in an experimental animal model.

Among the four M. mycoides subsp. *capri obg* subclones (43.7.3E, 43.7.4B, 9.2.1A, and 9.2.3B) that were characterized as described above, subclone 9.2.3B, which carried 3 mutations in the *obg* gene and showed the strongest TS^+^ phenotype, was selected for subsequent *in vivo* testing of attenuation using a caprine infection model.

**FIG 4:**
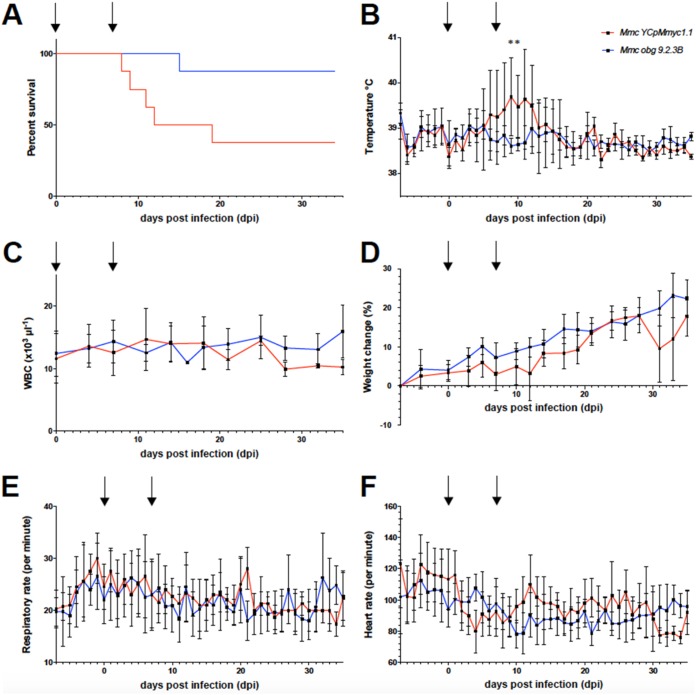
Survival rates and clinical parameters measured during *in vivo* testing of M. mycoides subsp. *capri obg* 9.2.3B (*obg* group) and M. mycoides subsp. *capri* YcpMmyc1.1 (control group). (A) The difference in survival rates (Kaplan-Meier curve) is significant according to both the Mantel-Cox test (*P* = 0.0351) and the Gehan-Breslow-Wilcoxon test (*P* = 0.0325). There were 8 goats per group. (B) Rectal temperature measured daily, shown as an average per group. The difference in average temperatures between the groups was significant on days 9 and 10 postinfection (day 9, *P* = 0.0006; day 10, *P* = 0.0053) using the Mann-Whitney test, coinciding with the peak of severity of disease in the group that received the M. mycoides subsp. *capri* YcpMmyc1.1 strain. (C) The white blood cell (WBC) count (measured twice weekly) was relatively stable throughout the trial, and no major difference between the groups was observed at any time point. (D) Both groups gained weight, measured three times weekly, continuously during the trial. (E) Respiratory rate, measured daily, remained within the normal range throughout the study. (F) Heart rate was measured daily and remained within the normal range for both groups. Arrows indicate days of infection (day 0, 10^8^ CFU per animal; day 7, 10^9^ CFU per animal). All graphs show the average measured parameters per group, with error bars showing SD.

Two groups of eight male goats, housed in separated rooms, were infected transtracheally with either M. mycoides subsp. *capri* YCpMmyc1.1 (22, 23) (control group) or M. mycoides subsp. *capri obg* subclone 9.2.3B (*obg* group). After two injections, one at 0 days postinfection (dpi), with 10^8^ CFU, and the other at 7 dpi, with 10^9^ CFU, animal health status was monitored daily by measuring key parameters, such as body weight, heartbeat, breathing frequency, and body temperature (Fig. 4). The following endpoint criteria for animal euthanasia were applied: a body temperature higher than 40.5°C for more than 3 consecutive days, the appearance of clinical signs indicating moderate to severe pain or distress, weight loss of more than 10% within 7 days, and a breathing frequency higher than 50 breaths · min^−1^ for more than 3 days. In the control group, 5 goats out of 8 met the endpoint criteria before the envisaged end of the trial (i.e., 35 dpi) and were euthanized in the course of the experiment, whereas only 1 goat (animal CM135) out of 8 met the same criteria in the *obg* group (Fig. 4A) (24). M. mycoides subsp. *capri* was cultured from 3 out of 8 animals that were infected with the control strain YCpMmyc1.1. The time points of M. mycoides subsp. *capri* isolation coincided with severe pyrexia. Bacterial titers ranged between 100 and 1,000 CFU · ml^−1^ (24). Animals that did not develop the disease had body temperatures ranging between 38°C and 39.5°C, which represents the physiological range for goats (Fig. 4B). Within the group infected with M. mycoides subsp. *capri* YCpMmyc1.1, those animals showing signs of disease developed fever (rectal temperature of >39.5°C) between days 3 and 10 after the first injection (Fig. 4B), and animal CM135 from the *obg* group showed fever starting at 13 dpi (data not shown). In most of the animals showing fever, body temperature reached 41°C in less than 3 days. In the *obg* group, the heart rate and breathing rate remained within the normal ranges for all animals, including animal CM135, throughout the study (Fig. 4E and F). We cultivated M. mycoides subsp. *capri* from the blood of the animals of the *obg* group, and only blood from the animal (CM135) that succumbed to disease gave a positive culture indicating bacteremia, with a titer of 10^3^ CCU · ml^−1^. Cultivation of M. mycoides subsp. *capri* from synovial fluid, urine, and pleural fluid of the same animal did not yield any positive cultures.

Necropsy of animals from the control group revealed severe and extensive necrosis, edema, and inflammation of the skin, subcutis, skeletal muscle of the neck, and the trachea around the site of inoculation in five animals. Histologically, there was extensive coagulation necrosis of these tissues surrounding the trachea in the vicinity of the inoculation site and a marked infiltration of neutrophilic granulocytes (Fig. S6a). These areas were surrounded by a rim of infiltration with mainly macrophages and lymphocytes. Regional lymph nodes showed purulent to necrotizing lymphadenitis, and there were multifocal neutrophilic infiltrates in the spleen. In one animal, there were multifocal, randomly distributed small areas of necrosis in neutrophilic infiltrates in the liver. These lesions were suggestive of septicemic spread of M. mycoides subsp. *capri* from the site of inoculation. Three animals of this group (animals CM045, CM047, and CM191) did not show macroscopic or histological lesions. Animal CM135 in the *obg* group had lesions similar to those in the control group (Fig. S6b). Overall, the data clearly demonstrated that even though one animal in the *obg* group developed disease and had to be euthanized, the virulence of M. mycoides subsp. *capri obg* subclone 9.2.3B was attenuated compared to its parental strain.

10.1128/mSphere.00030-19.7 FIG S6 Histopathological analyses. Representative histopathological sections from soft tissue of the neck close to the inoculation site depict large areas of necrosis and inflammation with neutrophilic granulocytes (asterisks) within the skeletal muscle (dots). (a) Tissue from animal CM154 inoculated with YCpMmyc1.1; (b) tissue from animal CM135 inoculated with M. mycoides subsp. *capri obg* subclone 9.2.3B with the reverted mutation from Asp85Gly to GAT (Gly85Asp); (c) tissue from an animal inoculated with M. mycoides subsp. *capri* GM12 during experimental challenge following infection with M. mycoides subsp. *capri obg* subclone 9.2.3B. Tissues were stained with hematoxylin and eosin. Size standards are displayed in each picture. Magnification, ×200. Download FIG S6, PDF file, 0.6 MB. Copyright © 2019 Lartigue et al. 2019 Lartigue et al. This content is distributed under the terms of the Creative Commons Attribution 4.0 International license.

### Genotypic analysis of the revertant iCM135.

In order to find out the reason for the loss of one animal in the *obg* group (Fig. 4A), the mycoplasma strain isolated from blood samples of the euthanized animal (iCM135 strain) was subjected to further analyses. Whole-genome sequencing showed that iCM135 carried a single reversion in one of the target mutations within the *obg* gene. Indeed, compared to the original TS^+^ M. mycoides subsp. *capri obg* subclone 9.2.3B, the GAT>GGT (Asp85Gly) triplets reverted back to GAT (Gly85Asp) in the iCM135 strain. The temperature sensitivity of iCM135 was analyzed *in vitro* using culture assays at 32°C or 40°C along with the M. mycoides subsp. *capri* control subclone 30.1.1 (intact *obg*). The experiment clearly showed that the iCM135 strain lost its temperature sensitivity and recovered a non-temperature-sensitive (TS^−^) phenotype that is similar to that of the parental strain (Fig. S7) and that the loss of the TS^+^ phenotype is probably due to the reversion observed at position 85 (Table S2). It should be stressed that a similar reversion of phenotype associated with reversion at position 85 was also observed *in vitro* during culture of subclone 9.2.1C at 40°C (Fig. S7).

10.1128/mSphere.00030-19.8 FIG S7 Analysis of the temperature sensitivity reversion phenotype during *in vitro* culture. In order to better understand the apparition of the reversion at position 85 in the sick animal of the *obg* group, subclone 9.2.1C, carrying the same genotype as the original subclone 9.2.3B, was cultured at 32°C and 40°C, and the apparition of the reversion was monitored by sequencing of the *obg* gene. The experiment was repeated four times. With this particular clone, the reversion was systematically detected at position 85 of the Obg protein when the samples were grown at 40°C (A9.2.1C_40_ subclone), while it was detected only once in the samples grown at 32°C after the same length of time (A9.2.1C_32_ subclone). Interestingly, the reversion that appeared *in vitro* was the same as the one that appeared *in vivo* in iCM135 (GGT reverted back to GAT [Gly85Asp]). Frozen stocks of A9.2.1C_32_ (no reversion) and A9.2.1C_40_ (reversion) were then cultured again at 32°C (A9.2.1C_32/32_ and A9.2.1C_40/32_) or 40°C (A9.2.1C_32/40_ and A9.2.1C_40/40_), along with the control strain 30.1.1 and the iCM135 strain. The results are presented in panel A (culture assays) and panel B (qPCR), detailed just below. Interestingly, subclone A9.2.1C_40_, exhibiting the reversion at position 85, showed a behavior almost identical to that of the iCM135 revertant and the control strain 30.1.1 at both temperatures. In contrast, subclone A9.2.1C_32_, not carrying the Gly85Asp reversion, retained its TS^+^ phenotype. These data suggested that the apparition of the reversion occurring at position 85 is selected at high temperature, leading to the loss of the TS^+^ phenotype. (Panel A) Growth monitoring of M. mycoides subsp. *capri obgmod* revertants at permissive (32°C) and nonpermissive (40°C) temperatures. This experiment required two phases of culture. In the first phase (not shown), M. mycoides subsp. *capri* revertants isolated *in vivo* (iCM135) were precultured in SP5 medium at 32°C along with the control subclone 30.1.1 (intact *obg*), M. mycoides subsp. *capri obgmod* subclone 9.2.1C (GAT>GGT [Asp85Gly], GGG>AGA [Gly124Arg], and TAT>TGT [Ile244Val]) and subclone 11.1 (GAT>AAC [Asp85Asn]). At pH 6.4, cell cultures were serially diluted (10^−1^ to 10^−9^) and incubated at either 32°C or 40°C. After 6 days, cell cultures (the last tube showing growth) were split into two parts: (i) 200 μl was collected for DNA extraction and sequencing of the *obg* region, and (ii) 800 μl was frozen. The sequencing data showed that position 85 of the *obg* gene reverted (Gly85Asp) in clone 9.2.1C when grown at 40°C but not at 32°C. These clones were renamed A9.2.1C_32_ and A9.2.1C_40_. In the second phase, frozen stocks (8 in total) were thawed, serially diluted twice (10^−1^ to 10^−9^) as described above, and incubated at either 32°C or 40°C for 8 consecutive days. Results are presented here. Pictures taken at 24 h and 48 h are shown. SP5 medium color changes from red to yellow were considered evidence of cell propagation, since glucose utilization during M. mycoides subsp. *capri* propagation causes an acid shift. Dotted rectangles indicate tubes (dilution, 10^−5^) from which the 100-μl aliquots were removed every 24 h from 0 h to 120 h to perform the qPCR analysis. (Panel B) qPCR assays. (Panel B, graph A) Bacterial concentrations of the different M. mycoides subsp. *capri obg* subclones were measured over time at 32°C (plain curves) and 40°C (dotted curves) up to 96 h using the above-described quantitative PCR assay. (Panel B, graph B) Samples previously incubated at 40°C (induction of reversion) were sequenced. Subclones that reverted on position 85 were incubated once again at 32°C (plain curves) or at 40°C (dotted curves), and bacterial titers were assessed. Error bars represent standard deviations from three independent replicates, all originating from a single culture for each subclone. Download FIG S7, PDF file, 0.5 MB. Copyright © 2019 Lartigue et al. 2019 Lartigue et al. This content is distributed under the terms of the Creative Commons Attribution 4.0 International license.

### Experimental challenge following infection with the 9.2.3B *obg* subclone.

**FIG 5:**
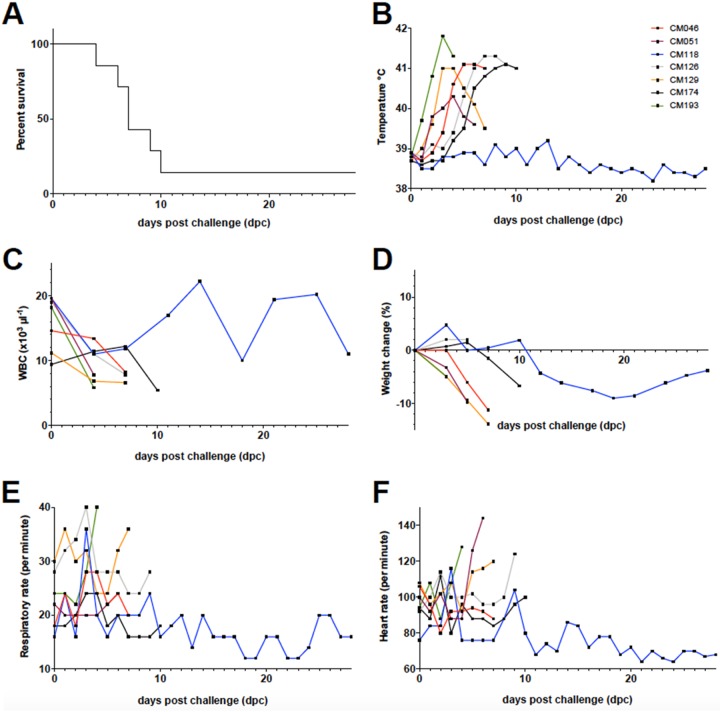
Experimental challenge of surviving goats following infection with the 9.2.3B *obg* subclone. Shown are survival rates and clinical parameters measured during *in vivo* challenge with wild-type M. mycoides subsp. *capri* GM12 (10^9^ CFU per animal) of the 7 animals that survived the initial infection with M. mycoides subsp. *capri obg* 9.2.3B. Due to the small number of animals and the heterogeneity of data, individual curves for clinical parameters are shown in panels B to F. (A) Six out of the seven animals were euthanized due to severity of disease between days 4 and 10 postchallenge. (B) All but one of the seven animals had fever (defined as a rectal temperature of >39.5°C), with peak temperatures ranging from 40.3°C to 41.8°C. One animal did not show an elevated temperature (animal CM118). (C) The white blood cell count dropped initially in 5 of the 7 animals but remained within the normal range throughout the study. (D) Weight loss was seen during severe disease in 5 animals. Goat CM118 lost weight after the other animals in the group had been euthanized but started to recover at around 20 days postchallenge. (E) Breathing frequency was within the normal range throughout the challenge experiment. (F) At the peak of severity of disease, the individual heart rates of animals CM193, CM051, CM129, and CM126 increased. The average heart rate remained within the normal range throughout the trial.

Since seven of eight goats infected with the 9.2.3B *obg* subclone survived after 35 dpi, we evaluated whether infection in these animals had triggered an immune response that would provide a certain degree of protection against an experimental challenge. The animals were challenged transtracheally with the M. mycoides subsp. *capri* GM12 strain (25). It should be noted that this strain is different from the M. mycoides subsp. *capri* YCpMmyc1.1 strain used as the control in the above-described experiment because it has not been submitted to a round of yeast cloning-genome transplantation followed by triple filter cloning. Therefore, the M. mycoides subsp. *capri* GM12 strain, at a low passage number, is known for its high pathogenicity, causing septicemia and death when administered at a high dose (27). At 0 days postchallenge (dpc), the seven animals were infected transtracheally with 10^9^ CFU of the M. mycoides subsp. *capri* GM12 strain, and animal health status was monitored daily for 28 days by measuring parameters such as body weight, heartbeat, breathing frequency, and body temperature (Fig. 5). Between 4 dpc and 10 dpc, 6 out of the 7 goats met the endpoint criteria and were euthanized, with only 1 animal (CM118) surviving until the end of the experiment (Fig. 5A). As expected with this highly pathogenic and septicemic strain of M. mycoides subsp. *capri*, infection resulted in a peak body temperature reaching ≥41°C for 5 out of the 6 animals that were euthanized. The M. mycoides subsp. *capri* challenge strain GM12 was isolated from the blood of 6 of the 7 animals. The bacterial titers ranged from 10^4^ to 10^10^ CFU · ml^−1^. The only animal for which the cultures remained negative was animal CM118, which survived until the end of the experiment, at 30 days postchallenge. Other clinical parameters were also affected although not uniformly among these animals (Fig. 5C to F).

Pathological lesions in 6 out of 7 of the M. mycoides subsp. *capri* GM12-inoculated animals were similar to those of the above-described control group, with massive necrosis and mainly neutrophilic inflammation in the soft tissues and the trachea of the neck extending from the inoculation site (Fig. S6c). All animals showed histopathological signs of septicemia, characterized as multifocal acute necrosis and neutrophilic infiltration in the spleen. In addition, three animals developed mild, purulent bronchopneumonia. Animal CM118 did not show any macroscopic or histological lesions.

## DISCUSSION

Using synthetic biology approaches, the essential gene *obg* encoding a P-loop GTPase was mutated on the M. mycoides subsp. *capri* genome, cloned in yeast, and subsequently transplanted into Mycoplasma capricolum recipient cells to obtain a set of M. mycoides subsp. *capri* mutants carrying various mutations. The targeted modifications of *obg* reproduced mutations that are known to be associated with a temperature-sensitive phenotype in various bacteria, including in a commercial live vaccine of M. synoviae.

Among the single *obg* mutations that were previously associated with a TS^+^ phenotype in E. coli, B. subtilis, and M. synoviae, the only one that showed a significant TS^+^ phenotype in M. mycoides subsp. *capri* is Gly80Glu (mutant 7.1) (Fig. 2). This mutation has been described in detail in Caulobacter crescentus and was also characterized by a marked TS^+^ phenotype associated with alterations in the cell cycle (21). In contrast, the single mutation described in the TS^+^ vaccine strain of M. synoviae (Gly124Arg) did not, by itself, produce a TS^+^ phenotype in M. mycoides subsp. *capri.* Recently, complementation of the Gly124Arg substitution in M. synoviae with a wild-type copy of the *obg* gene did not result in a TS^−^ phenotype (10), but some of the growth parameters, including growth rate and cell viability, were at least partially restored; it should be stressed that in this case, complemented clones expressed both wild-type Obg and the protein with the Gly124Arg substitution. In the *obg* mutants with more than one mutation, the complex interplay among them is probably due to structure constraints. For example, the reversion at position 85 (Gly85Asp) was associated with the loss of the TS^+^ phenotype in the 9.2.3B mutant, although the single mutation Asp85Asn (mutant 11.1) did not produce a significant TS^+^ phenotype in M. mycoides subsp. *capri*. As the replacement at this position has been done using two different amino acids (Gly and Asn), we cannot exclude the possibility that one change is more drastic than the other one regarding the growth of M. mycoides subsp. *capri* at a nonpermissive temperature.

The set of mutants that was obtained in this study finally resulted in a range of TS^+^ phenotypes. The mutant that was the most severely affected in its growth at 40°C was selected because the multiple mutations (at least the Asp85Gly and the Gly124Arg mutations) were considered to confer a higher level of safety against possible genetic reversion. These two mutations did not prove to be sufficient, since a single reversion at position 85 (Gly85Asp) was associated with the loss of the TS^+^ phenotype both *in vivo* (mutant iCM135) and *in vitro* (subclone A9.2.1C_40_). This indicates that a structural change in the protein that leads to the TS^+^ phenotype occurs only when both of the two mutations (Gly85Asp and Gly124Arg) are present, possibly together with the third mutation (Ile224Val). Therefore, the reversion in one of them was enough to lose the TS^+^ phenotype in M. mycoides subsp. *capri.* Obviously, the reversion of these mutations is a problem if one envisages obtaining safe vaccine strains with this approach. In fact, this reversion to the TS^−^ phenotype is already known for the vaccine strain of M. synoviae that was produced by chemical mutagenesis, but this attenuation is most likely due to other mutations in other genes, such as the ones encoding the oligopeptide permease (Opp) system (6, 10, 26).

A novel caprine challenge model has been established for M. mycoides subsp. *capri* GM12 (24, 27), which we basically followed, with slight modifications, as reported recently (24). This challenge model proved to be robust and reproducible (24, 27). Another caprine challenge model using an atypical M. mycoides subsp. *capri* strain was reported elsewhere (28), but as the number of infected animals was small and the outcome differed between groups markedly, we opted not to follow this challenge model. The model of infection performed here is a significant advancement, as there is a general lack of animal models for studying mycoplasma pathogenicity, which is mostly due to the high host specificity of mycoplasmas. However, this animal model has its own advantages as well as limits. Its main advantage is that it allows evaluation of the attenuation of mutants when derived from a strongly pathogenic strain such as M. mycoides subsp. *capri* GM12. Within a few days, the survival curves (Fig. 4) together with the data on bacteremia provide the main tools for evaluation of the outcome of infection. The weight of each clinical parameter, including body temperatures, is more difficult to evaluate because we used outbred goats that are genetically heterogeneous. In addition, as shown in this study, the high dose of infection with mutants provides the possibility of detecting pathogenic revertants. However, the limits of this animal model also need to be recognized. The transtracheal application of the 9.2.3B *obg* subclone was selected for comparison of virulence with the parental strain YCpMmyc1.1. This route was not meant to be used as prospective immunization route. However, we opted to challenge the 7 goats that survived the first phase of the experiment (35 dpi) because we expected the induction of an immune response, and it provides a comparison with similar results obtained with a GM12 *glf* mutant defective in capsule biosynthesis (24). Whereas 3 of the 8 animals infected with the *glf* mutant survived, only 1 of the 7 animals infected in this study with the *obg* mutant survived. This difference is not significant. It is difficult to discuss much more about this difference without a full analysis of the immune response in individual animals; our preliminary attempts using enzyme-linked immunosorbent assays (ELISAs) and immunoblotting did not provide meaningful results because of the high background of antibodies cross-reacting with M. mycoides subsp. *capri* antigens in the goats. This is likely due to the exposure of these animals to a number of parasites before being selected for this trial. Finally, it should also be stressed that very little is known about the immune determinants that would provide protection from challenge with highly virulent M. mycoides subsp. *capri*.

Until now, the tools from synthetic biology applied to *Mycoplasma* species have been mostly limited to building a minimal cell (20). However, we have shown that these tools are also useful for the elucidation of gene functions (29) and for the deciphering of host-pathogen interactions (30). The versatility of these approaches that combine in-yeast genome engineering and back-transplantation into a recipient cell is further demonstrated in the present study by showing our ability to generate targeted point mutations within the essential *obg* gene. Such an achievement was previously impossible using the genetic tools available for any *Mycoplasma* species. Since it is now possible to use synthetic biology tools for most of the ruminant-pathogenic *Mycoplasma* species that are closely related to M. mycoides subsp. *capri* (31), new strategies of vaccine design based on precise and multiple targets using genome engineering have become fully realistic. Within this global aim, the TS^+^ phenotype resulting from *obg* mutations described here is of interest if combined with (i) other mutations that hamper completely the possibility of reversion to a virulent phenotype and (ii) other deletion(s), such as deletion of the nonessential *glpO* gene known to be involved in the production of H_2_O_2_ (32). Owing to this new animal model, we now have the possibility of evaluating the attenuation of virulence of different mutants resulting from genome engineering approaches and the immune response providing protection against an experimental challenge.

## MATERIALS AND METHODS

### Bacterial and yeast strains and culture conditions.

Competent E. coli cells (ElectroMAX DH10B; Invitrogen) [F*^−^ mcrAΔ*(*mrr-hsdRMS-mcrBC*) ϕ80d*lacZΔ*M15 *ΔlacX74 recA1 endA1 araD139* Δ(*ara leu*)*7697 galU galK* λ− *rpsL nupG*] served as the host for cloning experiments and plasmid propagation. E. coli cells transformed with plasmids were grown at 37°C in Luria-Bertani (LB) broth or agar medium supplemented with 100 μg · ml^−1^ of ampicillin.

Wild-type Mycoplasma mycoides subsp. *capri* strain GM12 (25) and other M. mycoides subsp. *capri* derivatives were cultured in SP5 medium as described previously (31). Tetracycline was added to the medium when needed at 5 μg · ml^−1^. For genome transplantation experiments, restriction-free Mycoplasma capricolum subsp. *capricolum* (McapΔRE) recipient cells (22) were grown at 30°C in superoptimal broth (SOB) supplemented with 17% (vol/vol) fetal bovine serum, 1% (wt/vol) glucose, 0.002% (wt/vol) phenol red, and penicillin at 0.5 μg · ml^−1^ (SOB^+^ medium) (33).

Saccharomyces cerevisiae strain VL6-48N (*MAT*α *trp1*-Δ*1 ura3*-Δ*1 ade2-101 his3-*Δ*200 lys2 met14 cir°*) (34) was cultured at 30°C in YPDA (yeast extract peptone dextrose adenine) medium (Clontech) according to a standard protocol (35). Yeast transformed with mycoplasma genomes was grown in minimal SD (synthetic defined) base medium (Clontech) complemented with histidine dropout supplement (Clontech) (SD-His medium).

### M. mycoides subsp. *capri* genome engineering in yeast.

The genome of the modified M. mycoides subsp. *capri* clone YCpMmyc1.1 (strain GM12) was previously cloned into S. cerevisiae strain VL6-48N (22). Targeted nucleotides corresponding to the amino acids at position 80, 85, or 124 of the M. mycoides subsp. *capri* Obg protein (Fig. 1A; see also Fig. S1 in the supplemental material) were mutated in the YCpMmyc1.1 genome cloned into yeast by using the TREC-IN (tandem repeat endonuclease cleavage-insertion) method as previously described (36). The experimental procedure used to obtain these engineered genomes is shown in Fig. 1B and detailed in the legend of Fig. S2 in the supplemental material (primers used during the procedure are listed in Table S1). At the end of the process, YCpMmyc1.1*-obg* genomes were obtained and contained mutations at either position 239/240, 253/255, or 369/371 of the *obg* gene, which corresponds to position 80, 85, or 124 of the Obg protein, respectively. To check this, total DNA was extracted from all selected yeast recombinants, and the DNA was analyzed by simplex PCR (Table S1), multiplex PCR, and pulsed-field gel electrophoresis (PFGE) as described previously (29) (Fig. S3). The presence of the mutations was confirmed by sequencing the 1,601-bp *obg* PCR fragment obtained using the primers obgF1 and obgR2 (Fig. S2). For multiplex PCR, a set of 11 different pairs of primers was used as previously described (22) (Table S1).

The TREC-IN method was also used to build a genome equivalent to the mutant 43.7 genome but with a repaired *nadE* sequence. Using the primer pair obgF1/obgR3 (Table S1) and mutant 43.7 genomic DNA, we amplified the *obg* gene with changes at positions 254, 369, 371, and 732 plus a 113-bp segment located upstream of the *obg* gene. This amplicon was then used as a template for PCR with primers cassette B-forward-F1 and cassette D-reverse-R1 to create a minicassette, named minicassette BC, with 60-bp overlaps with minicassette A at its 5′ end and 60-bp overlaps with the sequence downstream of 5′ Kan (positions 1 to 515) of the YCpMmyc1.1*-Δobg*::*URA3* genome, at its 3′ end. Minicassettes BC and A were finally cotransformed into VL6-48N yeast clone 34 harboring the YCpMmyc1.1*-Δobg*::*URA3* genome (step 2 of the TREC-IN method). The next steps of the TREC-IN method as well as all genotypic tests were performed as described above.

### Back-transplantation of the engineered YCPMmyc1.1*-obgmod* genomes into mycoplasma recipient cells to produce the M. mycoides subsp. *capri obg* mutants.

In-yeast-engineered M. mycoides subsp. *capri* genomes were isolated in agarose plugs and transplanted back into restriction-free M. capricolum subsp. *capricolum* (McapΔRE) recipient cells as previously described (22, 37). The resulting transplants were selected on SP5 medium plates supplemented with 5 μg · ml^−1^ of tetracycline. After 3 successive passages in liquid medium, transplants were analyzed by PCR using M. mycoides subsp. *capri*-specific primers (Table S1), multiplex PCR (Table S1), and PFGE, as previously described (22). The *obg* gene was systematically amplified using the primer pair Obg-F1/ObgR1 (Fig. S2B) and sequenced in order to verify the presence of the desired mutations.

Mutants showing temperature-sensitive phenotypes were submitted to three successive rounds of filter cloning using 0.22-μm sterilizing-grade filters (38). Subclones were once more assessed for their temperature-sensitive properties, and their whole genomes were sequenced using Illumina next-generation sequencers (see the supplemental material). Mutant and subclone names and main characteristics are summarized in Table 1.

### Assessment of temperature sensitivity by serial dilution and qPCR.

Mutants and subclones were grown at 32°C in SP5 medium until the pH reached 6.4. The temperature sensitivity of the samples was then analyzed by serial dilutions (10^−1^ to 10^−9^) of the cell cultures in SP5 medium, followed by an incubation period of 8 days at a permissive temperature (32°C) or a nonpermissive temperature (40°C). Culture color change was monitored daily and compared to that of a culture of WT M. mycoides subsp. *capri* strain GM12 grown under the same conditions. The cell concentration of each initial culture (pH 6.4) was determined by plating dilutions on SP5 plates. Just before putting the samples into the incubator (*T*_0_) and then every 24 h during 6 days (*T*_24_ to *T*_144_), a 100-μl aliquot was taken from the “10^−8^ dilution tube” (for the mutants) and from the “10^−5^ dilution tube” (for the subclones) for qPCR assays. These qPCR assays were performed using a protocol described previously (31). Briefly, qPCR measurements were conducted in 96-well plates using the SsoFast EvaGreen supermix (Bio-Rad) on the LightCycler 480 real-time PCR system (Roche) according to the manufacturers’ instructions. Standard curves used to quantify the numbers of M. mycoides subsp. *capri* genomes were run independently for each assay by using purified genomic DNA (Wizard genomic DNA purification kit; Promega), with samples containing between 10^2^ and 10^8^ genomes. Measurements were done in triplicates for all samples. Data analyses were performed using GraphPad Prism statistical software version 7. All qPCR data were previously log transformed to meet model assumptions, and the Benjamini-Hochberg method (39) was used to adjust the false discovery rate (FDR) associated with the test. Analyses were done using an unpaired (independent) *t* test to compare the concentrations (means) of the different mutants with those of the M. mycoides subsp. *capri* controls (30.1 or 30.1.1) at each time point.

### Animal studies.

The animal experiments were performed under strict ethical rules (see “Ethics statement,” below). Briefly, 16 male crossbred goats (Capra hircus), 1 to 2 years of age, were randomly selected from the International Livestock Research Institute (ILRI) ranch in Kapiti (see the supplemental material for details). Five days before experimental infection, all animals were transferred to the animal biosafety level 2 (ABSL2) unit and split randomly into two groups of eight animals (positive-control and *obg* groups), and each group was housed in a separate room. On day 0 postinfection (p.i.) and day 7 p.i., goats of the positive-control group and the *obg* group were infected transtracheally (the injection site was 5 to 10 cm distal from the larynx) with M. mycoides subsp. *capri* YCpMmyc1.1 and M. mycoides subsp. *capri* YCpMmyc1.1-*obg*, respectively. On day 0 p.i., each animal was infected with a 1-ml liquid culture containing 10^8^ CFU, followed by a flush with 5 ml of phosphate-buffered saline (PBS); on day 7 p.i., this procedure was repeated, except that each animal was infected with a liquid culture containing 10^9^ CFU. The animals were allowed to move freely within the ABSL2 unit and received hay and water *ad libitum* as well as pellets in the morning. Heart rate, breathing frequency, rectal temperature, and oxygen blood saturation were measured daily in the morning hours. Animals showing either a body temperature of >40.5°C for >3 consecutive days, moderate to severe pain or distress, weight loss of >10% within 7 days, or a breathing frequency of >50 breaths/min for >3 days were euthanized. More information regarding the group infected with YCpMmyc1.1 were described previously by Jores et al. (24).

### Ethics statement.

All protocols of this study were designed and performed in strict accordance with Kenyan legislation for animal experimentation and were approved by the institutional animal care and use committee (IACUC) (reference no. IACUC-RC2016-04, signed by Roger Pelle). Since 1993, the ILRI has complied voluntarily with the United Kingdom’s Animals (Scientific Procedures) Act 1986 (http://www.homeoffice.gov.uk/science-research/animal-research/), which contains guidelines and codes of practice for the housing and care of animals used in scientific procedures. The study reported here was carried out in strict accordance with the recommendations in the standard operating procedures of the ILRI IACUC (ILRI IACUC ref no. 2016.06) and with adequate consideration of the 3 R’s (replacement of animal with nonanimal techniques, reduction in the number of animals used, and refinement of techniques and procedures that reduce pain and distress). As this project also received financial support from the NSF (NSF proposal IOS-1110151 entitled BREAD: Toward Development of a Vaccine for Contagious Bovine Pleuropneumonia [CBPP]) awarded to the J. Craig Venter Institute (JCVI), the protocols of this study were also approved by the JCVI’s own IACUC. The project was approved initially by the IACUC on 17 August 2011, reapproved on 6 October 2014 (no. 011-14), and signed by Mark Adams, JCVI Scientific Director.

### Pathomorphological and histological analyses.

Complete necropsy was performed on all animals. Tissue samples of the neck region around the inoculation site and all internal organs were fixed in 10% buffered formalin for 72 h and subsequently routinely processed for paraffin embedding. Tissue sections were cut at 3 μm, stained routinely with hematoxylin and eosin (H&E), and evaluated by a board-certified pathologist.

### Data availability.

We declare that the data supporting the findings of this study are available within the paper and its supplemental material. The sequences from this study are available from the NCBI under SRA accession no. PRJNA510711.

10.1128/mSphere.00030-19.1 TEXT S1 Supplemental methods for animal studies. Download Text S1, DOC file, 0.04 MB. Copyright © 2019 Lartigue et al. 2019 Lartigue et al. This content is distributed under the terms of the Creative Commons Attribution 4.0 International license.
