# Supplementary material for: Attenuation of a Pathogenic Mycoplasma Strain by Modification of the obg Gene by Using Synthetic Biology Approaches
Source: mSphere. 2019 May 22;4(3):e00030-19. doi: 10.1128/mSphere.00030-19 (PMC6531878; doi:10.1128/mSphere.00030-19)
Supplement: FIG S1 [file mSphere.00030-19-sf001.pdf]

A -

N-terminal domain

```
Bsub    --MFVDQVKVYVKGGDGGNGMVAFRREKYVPKGGPAGGDGGKGGDVVFEVDEGLRTLMDF 58
Ecoli   -MKFVDEASILVVAGDGGNGCVSFRREKYIPKGGPDGGDGGDGGDVWMEADENLNTLIDY 59
Ttherm  --MFQDVLVITVAAGRGGDGAVSFRREKFVPKGGPDGGDGGRGGSVYLRARGSVDSLSRL 58
MsTS-   MAKFIDQVKIMLKAGKGGDGMISFRREAHVDKGGPDGGDGGTGGNIYFVADLGKNTLLSF 60
MsTS+   MAKFIDQVKIMLKAGKGGDGMISFRREAHVDKGGPDGGDGGTGGNIYFVADLGKNTLLSF 60
Mmc     -MKFVDFADLIIKAGKGGDGAVSFLHALFVPNGGPNGGDGGDGGSVYFLGDEGKHSLLDL 59
          * *   : : .* **:* ::* :  .: :*** ***** **.: :    .  :*

                            80    85
Bsub    RYKKHFKAIRGEHGMSKNQHGRNADDMVIKVPPGTVVTDDDTKQVIADLTEHGQRAVIAR 118
Ecoli   RFEKSFRAERGQNGASRDCTGKRGKDVTIKVPVGTRVIDQGTGETMGDMTKHGQRLLVAK 119
Ttherm  S-KRTYKAEDGEHGRGSQQHGRGGEDLIIEVPRGTRVFDADTGELLADLTEEGQTVLVAR 117
MsTS-   YKNKFIIAEDGVKGGPKNLYGAKGKDTIVKVPLGTLVYKNKK--IVADVIKENHLYLVAK 118
MsTS+   YKNKFIIAEDGVKGGPKNLYGAKGKDTIVKVPLGTLVYKNKK--IVADVIKENHLYLVAK 118
Mmc     KLQKKYSAQDGFKGDIKNMHGAKGEDKIIKVPVGTILYDKKTNTILADINENNKLVLIAK 119
          ::  *  * :*   :  *  ..*  ::** ** : .  .   :.*: :.:  ::*:

           124                                           G1
Bsub    GGRGGRGNSRFATPANPAPQLSENGEPGKERYIVLELKVIADVGLVGFPSVGKSTLLSVV 178
Ecoli   GGWHGLGNTRFKSSVNRTPRQKTNGTPGDKRELLLELMLLADVGMLGMPNAGKSTFIRAV 179
Ttherm  GGAGGRGNMHFVSPTRQAPRFAEAGEEGEKRRLRLELMLIADVGLVGYPNAGKSSLLAAM 177
MsTS-   GGKGGRGNNKFKTSKNTAPRIAENGMPGEKYEANIVLKIISDVGLVGLPSCGKSTLINAL 178
MsTS+   GGKGRRGNNKFKTSKNTAPRIAENGMPGEKYEANIVLKIISDVGLVGLPSCGKSTLINAL 178
Mmc     GGKGGKGNARFANSRNKAPTIFEAGELGQEFEIRAELKVIADVGFVGLPNAGKSTLLRAI 179
        **   ** :* .. . :*     *  *.:      *  ::***::* *. ***::: .:

           Switch I                         Switch II
             G2                       G3
Bsub    SSAKPKIADYHFTTLVPNLGMVETDDGRSFVMADLPGLIEGAHQGVGLGHQFLRHIERTR 238
Ecoli   SAAKPKVADYPFTTLVPSLGVVRMDNEKSFVVADIPGLIEGAAEGAGLGIRFLKHLERCR 239
Ttherm  TRAHPKIAPYPFTTLSPNLGVVEVSDEERFTLADIPGIIEGASEGKGLGLEFLRHIARTR 237
MsTS-   SNAKAKVAEYEFTTLVPQLGLVKYYDY-SYTIADLPGLIKGASLGKGLGIQFLRHIERCK 237
MsTS+   SNAKAKVAEYEFTTLVPQLGLVKYYDY-SYTIADLPGLIKGASLGKGLGIQFLRHIERCK 237
Mmc     SNSKPVVADYPFTTITPQLGVARTKNNDTFIVADLPGLIQGASLGKGLGHQFLKHIERCL 239
        : ::. :* * ***: *.**:..  :   : :**:**:*:**  * *** .**:*: *
```

GTP-binding domain

```
           244                                        G4
Bsub    VIVHVIDMSG-LEGRDPYDDYLTINQELSEYNLRLTERPQIIVANKMDMP---EAAENLE 294
Ecoli   VLLHIIDIDP-IDGTDPVENARIIISELEKYSQDLATKPRWLVFNKIDLLDKVEAEEKAK 298
Ttherm  VLLYVLDAA-----DEPLKTLKTLRKEVGAYDPTLLRRPSLVALNKVDLL----EEEVVK 288
MsTS-   VVIHIVDFGS--PDKDSIQSYETIQKELESYKLNLTQKPQLVVANKSDLA---NFKNNI- 291
MsTS+   VVIHIVDFGS--PDKDSIQSYETIQKELESYKLNLTQKPQLVVANKSDLA---NFKNNI- 291
Mmc     VICHIIDASGNFGSEDIIKNYELIRDELKAYNLNLEKRPEIIVLNKMDLD---EAQLNLL 296
        *: :::*         :  .    : .*:  *.  *   :*  :. ** *:

                   G5
Bsub    AFKEKLTDD-YPVFPISAVTREGLRELLFEVANQLENTPEFPLYDEEELTQNRVMYTMEN 353
Ecoli   AIAEALGWE-DKYYLISAASGLGVKDLCWDVMT----------------------FIIEN 335
Ttherm  ALADALAREGLAVLPVSALTGAGLPALKEALHALVRSTP-----------PPEMPKPVPR 337
MsTS-   -EKFKAKYPNIEIIEISALNYHNVENLKKIIWEFLEKTSHLQIKIED--DFETEVKEINY 348
MsTS+   -EKFKAKYPNIEIIEISALNYHNVENLKKIIWEFLEKTSHLQIKIED--DFETEVKEINY 348
Mmc     DEKIINYFKDKKVVRISGLKKENIDQLLFMIYEELKVAKKQPLWELDKNNDQDEIAIYKF 356
                    :*. .  .:  *  :
```

C-terminal domain

```
Bsub    EEVPF--NITRDPDGVFVLSGDSLERLFKMTDFSRDESVKRFARQMRGMGVDEALRERGA 411
Ecoli   PVVQA--EEAKQPEKVEFMWDDYHRQQLEEIAEEDDE------------DWDDDWDE-DD 380
Ttherm  KEVQAGVEVVPVAEGVYEVRAPEVERYLARIKGDLMEAAGYLQEVFRRHGVEAALRAKGV 397
MsTS-   EP---DFVIQKMNRNHFKVTGKKIEELVLKIPINTFDNLMRFNNILKKIGVWETLIKRDI 405
MsTS+   EP---DFVIQKMNRNHFKVTGKKIEELVLKIPINTFDNLMRFNNILKKIGVWETLIKRDI 405
Mmc     EEQKEDIQVYNKGNNRWEIAGETIFKIYQKFPIWTEDNLLMFNEKLKETGVYETLVKKGI 416
                         :      .         :          .       .

Bsub    KDGDIIRLLEFEFEFID-- 428
Ecoli   EEG-----VEFIYKR---- 390
Ttherm  RAGDLVRIGGLEFEYIPEV 416
MsTS-   QKGDLVEIYQHKFEWEEEL 424
MsTS+   QKGDLVEIYQHKFEWEEEL 424
Mmc     KKGDFVKVFDYELEWTD-- 433
        . *          :
```

B -

Percentage of identity	*Mmc* Whole aa sequence	*Mmc* Obg N-term part	*Mmc* Obg GTP binding domain	*Mmc* Obg C-term part		*Mmc* Obg N-term part + GTP binding domain
M. synoviae	44.89	51.25	48.72	34.69		50
B. subtilis	41.82	47.83	51.85	17.14		49.85
E. coli	41.87	43.21	45.68	22		44
T. thermophilus	37.7	46.25	41.40	17.71		43.85
