# Supplementary material for: Attenuation of a Pathogenic Mycoplasma Strain by Modification of the obg Gene by Using Synthetic Biology Approaches
Source: mSphere. 2019 May 22;4(3):e00030-19. doi: 10.1128/mSphere.00030-19 (PMC6531878; doi:10.1128/mSphere.00030-19)

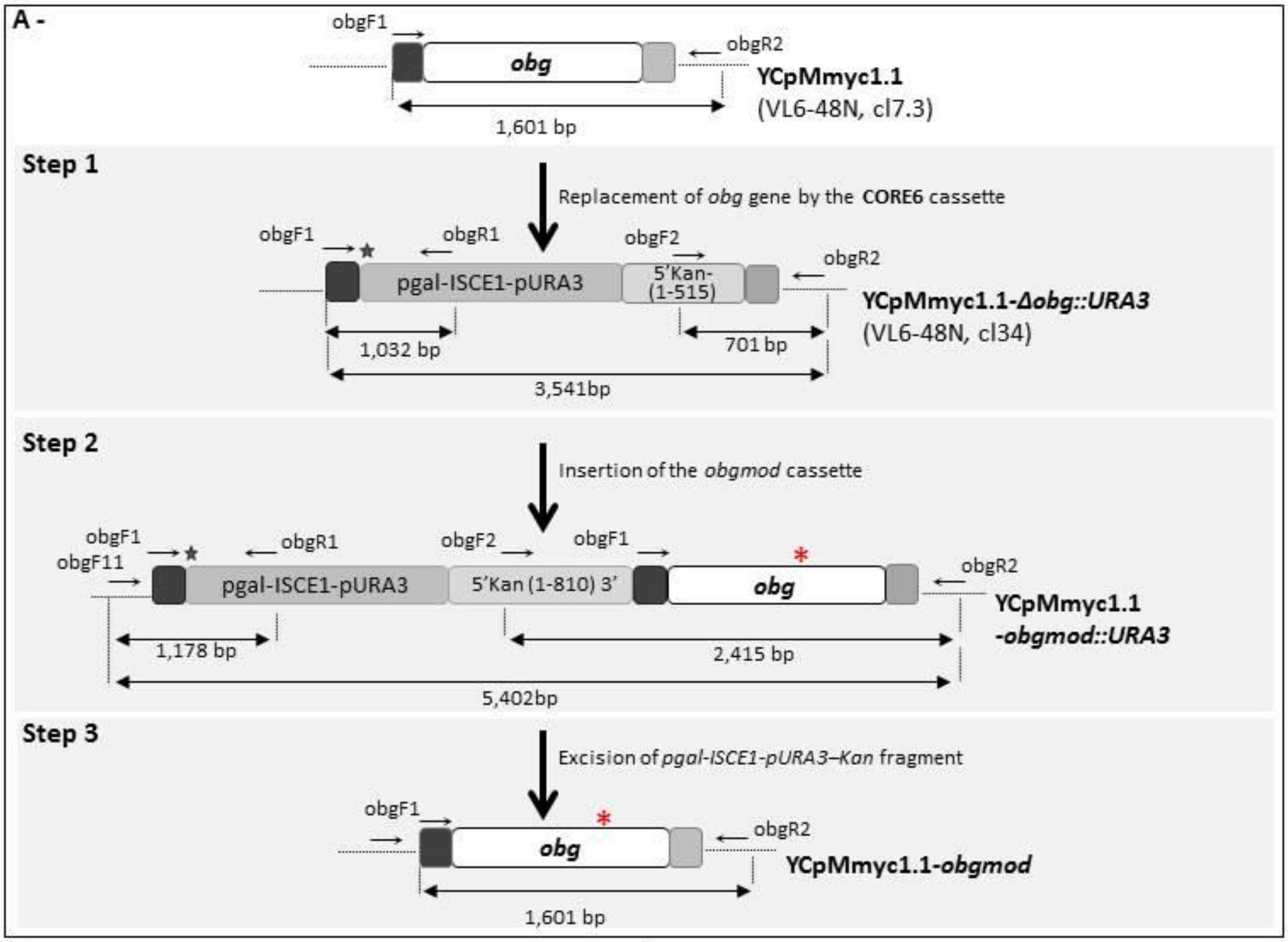

B -

Primers	Sequence (5′→ 3′)	Tm
Obg-F1	Atg tga tga gtg ttg aaa tac	56
Obg-F11	Caa tct cca att cca att gga tc	60
Obg-R2	Gaa taa att ggc att cat aca g	58
Obg-R1	Cca tca tcc atg aac cag tat g	54
Obg-F2	Atg ggt aag gaa aag act cac	60

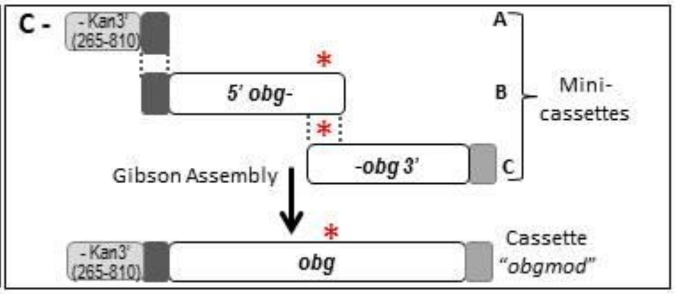

Supplement: FIG S2 [file mSphere.00030-19-sf002.pdf]
