# Supplementary figures and images for: Attenuation of a Pathogenic Mycoplasma Strain by Modification of the obg Gene by Using Synthetic Biology Approaches
Source: mSphere. 2019 May 22;4(3):e00030-19. doi: 10.1128/mSphere.00030-19 (PMC6531878; doi:10.1128/mSphere.00030-19)

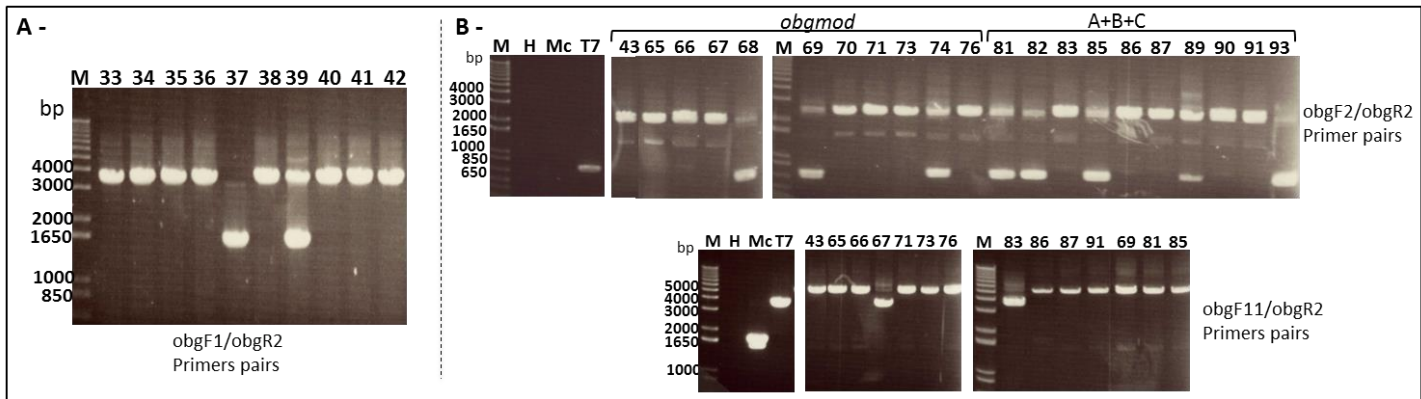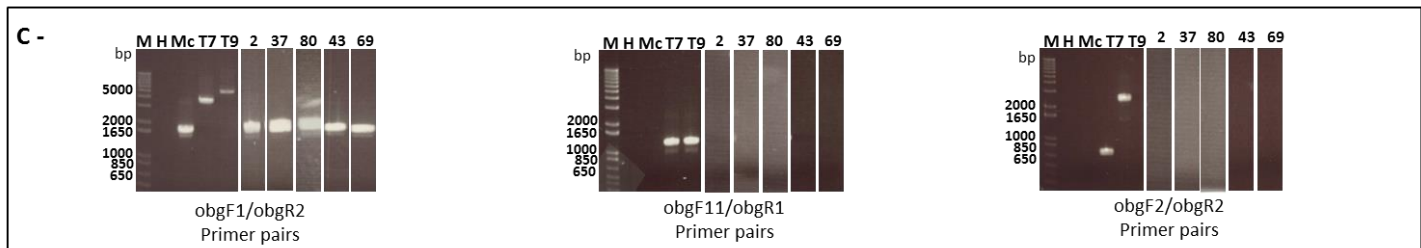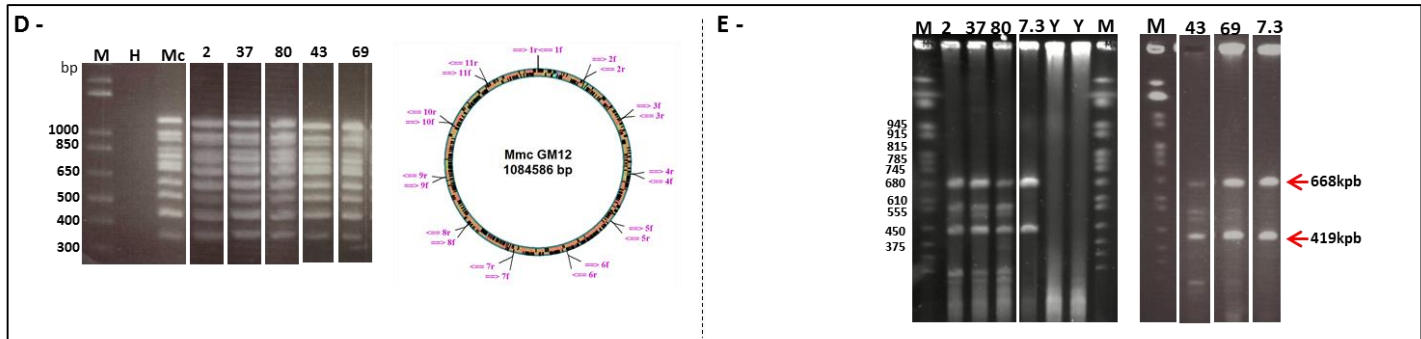

Supplement: FIG S3 [file mSphere.00030-19-sf003.pdf]

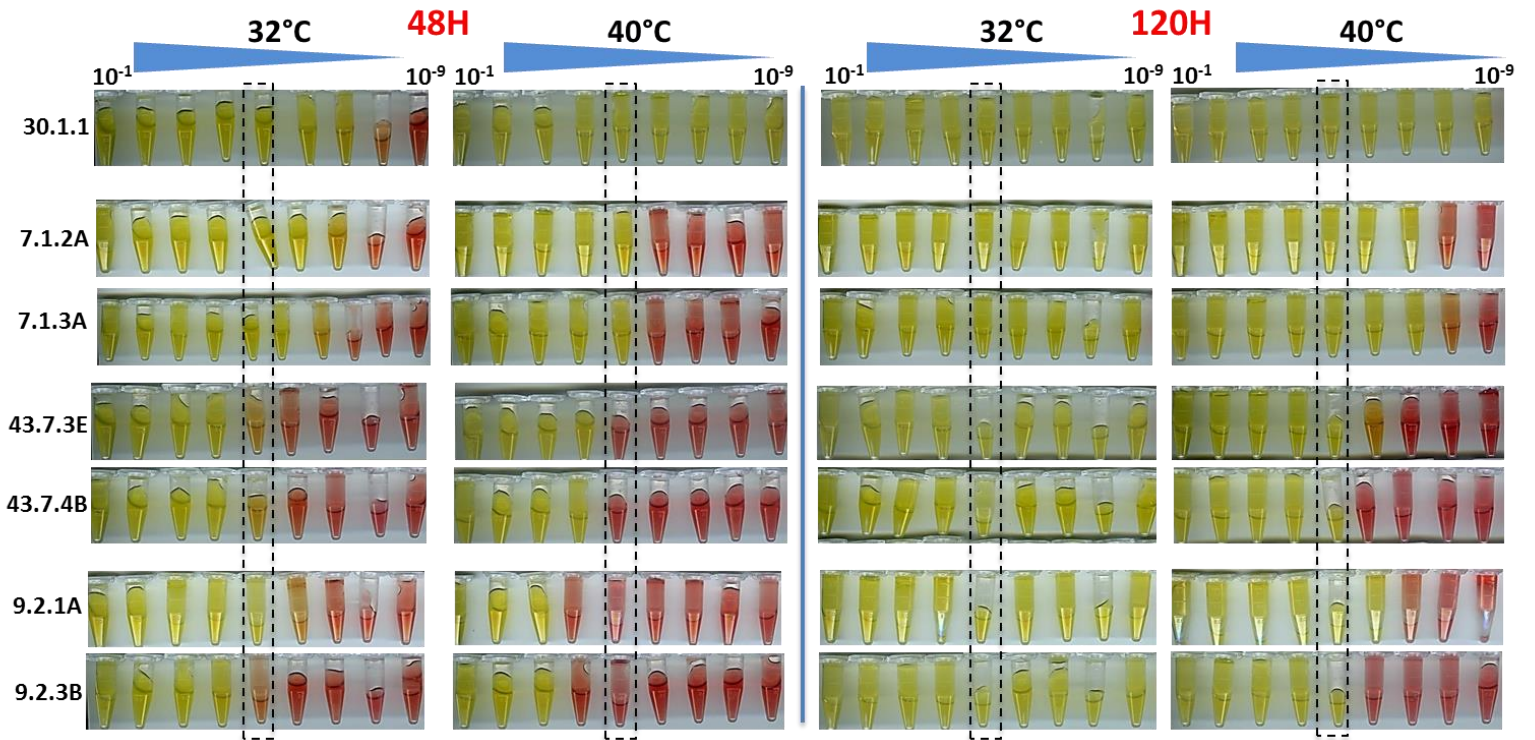

Supplement: FIG S5 [file mSphere.00030-19-sf005.pdf]

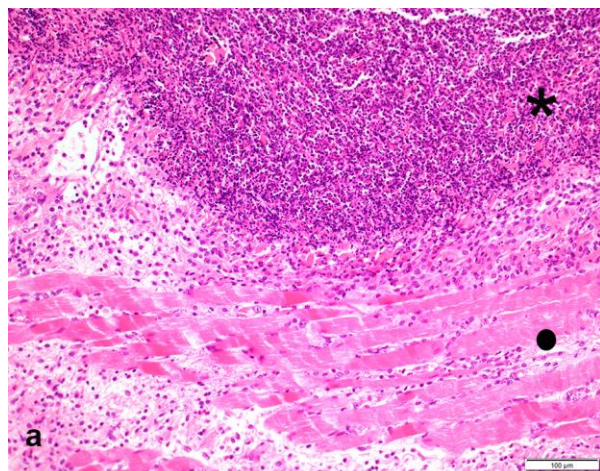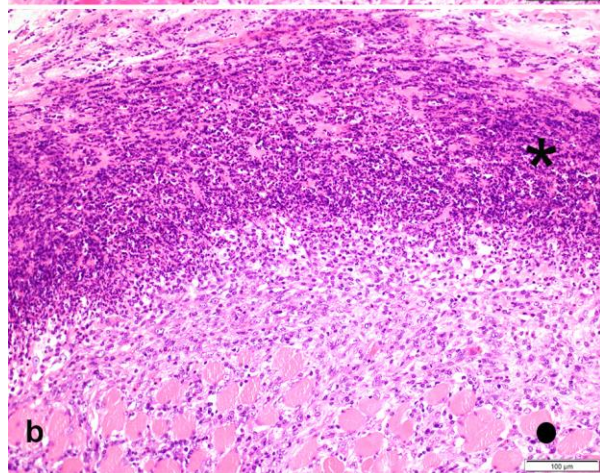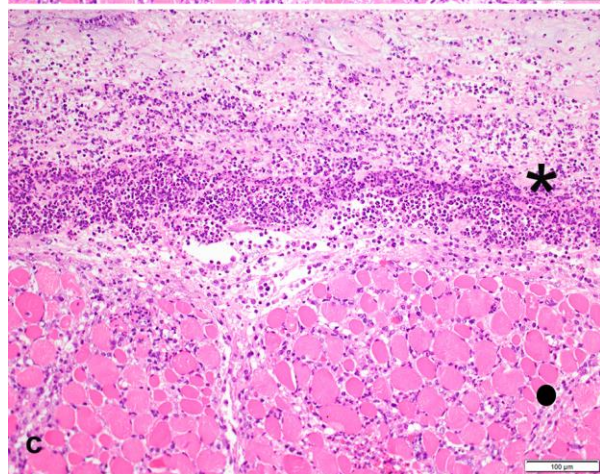

Supplement: FIG S6 [file mSphere.00030-19-sf006.pdf]

Panel A

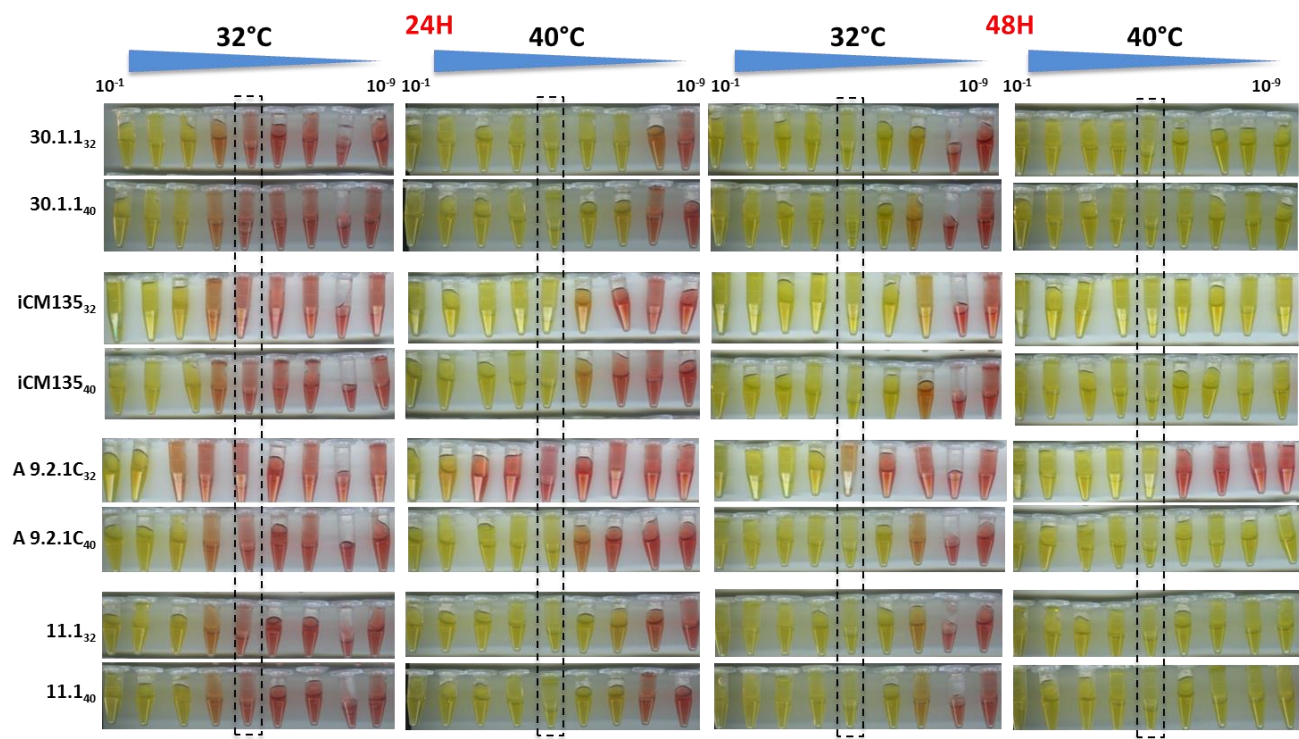

Panel B

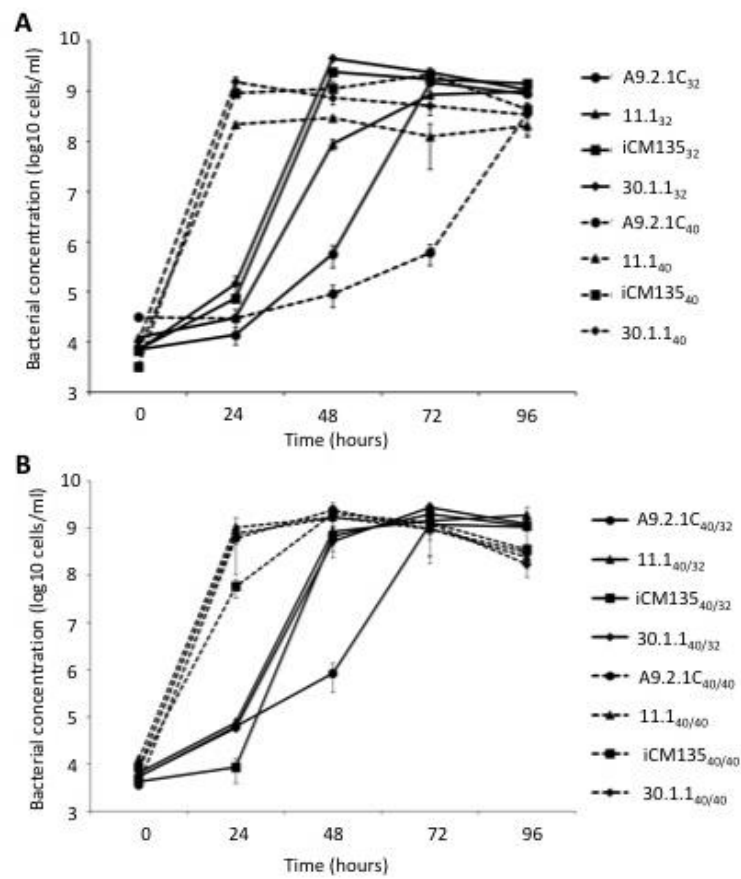

Supplement: FIG S7 [file mSphere.00030-19-sf007.pdf]
