# Supplementary material for: Attenuation of a Pathogenic Mycoplasma Strain by Modification of the obg Gene by Using Synthetic Biology Approaches
Source: mSphere. 2019 May 22;4(3):e00030-19. doi: 10.1128/mSphere.00030-19 (PMC6531878; doi:10.1128/mSphere.00030-19)
Supplement: TEXT S1 [file mSphere.00030-19-s0001.doc]

**Supplementary methods concerning the animal experiments**

Sixteen male crossbred goats (*Capra hirca*), 1-2 years of age, randomly selected from the ILRI ranch in Kapiti (a region not reported of infections with *Mycoplasma* belonging to the *M. mycoides* cluster), were transferred to the ILRI campus in Nairobi and kept in quarantine for 1 month. After arrival at the campus, all animals were dewormed twice using Levamisole Hydrochloride 3.0% w/v and Oxyclozanide 6.0% w/v (Levafas, Norbrook Kenya Ltd) and treated prophylactically against babesiosis and anaplasmosis using imidocarb (Imizol, Shring-Plough Animal Helath, USA). The goats were vaccinated against Peste des petits ruminants (PPR) (Pestivax, Kevevapi, Kenya), blackleg (Blanthax vaccine, Cooper, Kenya) and foot and mouth disease (Fotivax, Kevevapi, Kenya). All animals were tested culturally negative for the presence of mycoplasma in the nasal cavity using nasal swabs.

Liquid mycoplasma cultures used to infect the animals were prepared as follows. Mycoplasmas were cultivated in PPLO medium supplemented with horse serum (Sigma, product number H 0146) to early logarithmic phase, aliquoted and stored at -80°C. The cfu was determined using one aliquot. Just before infection the vials were thawed, the volume was adjusted to 1 ml and injected transtracheally followed by 5 mL of PBS solution. A short coughing reflex of the animals afterwards confirmed the correct delivery of the inoculum into the tracheal lumen. Following the experimental infection, three veterinarians monitored the health status of the animals throughout the experiment. Heart rate, breathing frequency, rectal temperature and oxygen blood saturation were measured daily in the morning hours. The latter two were measured with the M750 digital thermometer (GLA Agriculture Electronics, USA) and VE H100B Veterinary Pulse Oximeter (EDAN, USA), respectively. The body weight (kg) was measured three times a week. Blood samples for subsequent analysis were taken twice a week by jugular vein puncture. The white blood cell (WBC) count of blood samples (EDTA-blood) was performed using Celltac α MEK-6450 (Nihon Kohden, Japan). The settings were adjusted to allow for measurement of caprine blood: WBC Sensitivity = 10, WBC Threshold = 7, RBC Sensitivity = 15, RBC Threshold: 3, RBC AUTO = OFF, PLT Threshold = 5. The blood samples were thoroughly mixed with an equal volume of buffer (ISOTONAC 3, Nihon Kohden, Japan) prior to measurement. The resulting values for WBC count were doubled prior to analysis to account for the buffer dilution. Analysis was done with the QP-821V Data Management Software LITE (Nihon Kohden, Japan). Nasal swabs were taken also twice a week and stored in liquid media at -80C°.
