# Supplementary material for: Attenuation of a Pathogenic Mycoplasma Strain by Modification of the obg Gene by Using Synthetic Biology Approaches
Source: mSphere. 2019 May 22;4(3):e00030-19. doi: 10.1128/mSphere.00030-19 (PMC6531878; doi:10.1128/mSphere.00030-19)
Supplement: FIG S4 [file mSphere.00030-19-sf004.pdf]

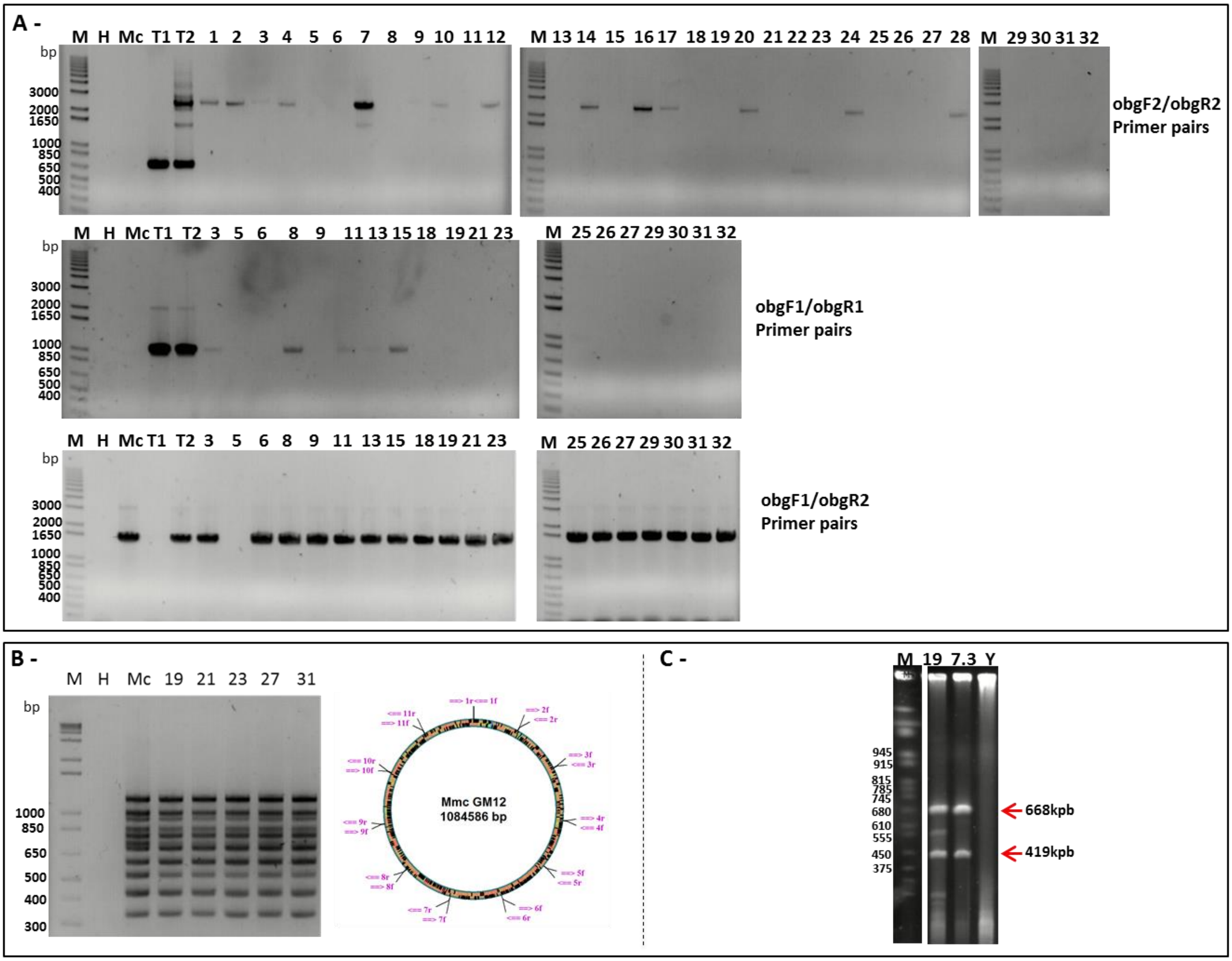
A -
M H Mc T1 T2 1 2 3 4 5 6 7 8 9 10 11 12
M 13 14 15 16 17 18 19 20 21 22 23 24 25 26 27 28
M 29 30 31 32
bp
3000
2000
1650
1000
850
650
500
400
obgF2/obgR2
Primer pairs
M H Mc T1 T2 3 5 6 8 9 11 13 15 18 19 21 23
M 25 26 27 29 30 31 32
obgF1/obgR1
Primer pairs
M H Mc T1 T2 3 5 6 8 9 11 13 15 18 19 21 23
M 25 26 27 29 30 31 32
obgF1/obgR2
Primer pairs
B -
M H Mc 19 21 23 27 31
bp
1000
850
650
500
400
300
Mmc GM12
1084586 bp
C -
M 19 7.3 Y
945
915
815
785
745
680
610
555
450
375
668kpb
419kpb
